# Supplementary material for: Efficient Synthesis of Imine-Carboxylic Acid Functionalized Compounds: Single Crystal, Hirshfeld Surface and Quantum Chemical Exploration
Source: Molecules. 2023 Mar 27;28(7):2967. doi: 10.3390/molecules28072967 (PMC10096040; doi:10.3390/molecules28072967)
Supplement: Supplementary file 1 [file molecules-28-02967-s001.zip › molecules-2253469-supplementary.pdf]

Efficient Synthesis of Imine-Carboxylic Acid Functionalized Compounds: Single crystal, Hirshfeld Surface and Quantum Chemical Exploration

Muhammad Nawaz Tahir,[1] Akbar Ali,*[2] Muhammad Khalid,*[3,4] Muhammad Ashfaq,[1] Mubashir Naveed,[1] Shahzad Murtaza, [3,4] Iqra Shafiq, [3,4] , Muhammad Adnan Asghar,[5] Raha Orfali,[6*] and Shagufta Perveen,[7]

[1]Department of Physics, University of Sargodha, 40100, Pakistan

[2]Department of Chemistry, Government College University Faisalabad, Faisalabad 38000, Pakistan.

[3]Department of Chemistry, Khwaja Fareed University of Engineering& Information Technology, Rahim Yar Khan, 64200, Pakistan

[4]Centre for Theoretical and Computational Research, Khwaja Fareed University of Engineering & Information Technology, Rahim Yar Khan, 64200, Pakistan

[5]Department of Chemistry, Division of Science and Technology, University of Education Lahore, Pakistan

[6]Department of Pharmacognosy, College of Pharmacy, King Saud University, PO Box 2457, Riyadh 11451, Saudi Arabia

[76]Department of Chemistry, School of Computer, Mathematical and Natural Sciences, Morgan State University, Baltimore, MD, 21251, USA

*Corresponding authors E-mail addresses: Dr. Akbar Ali: akbarali@gcuf.edu.pk; Muhammad.khalid@kfueit.edu.pk; rorfali@ksu.edu.sa

Table S1: SC-XRD experimental details of **HMBA, DHBA**.

**Crystal data**	**HMBA**	**DHBA**
CCDC	2117442	2117443
Molecular formula	$C_{16}H_{17}NO_5$	$C_{16}H_{15}I_2NO_4S$
M_r	303.30	571.15
Crystal system, space group	Orthorhombic, $P2_12_12_1$	Monoclinic, $P2_1/c$
Temperature (*K*)	296	296
a, b, *c* (Å)	4.7768 (11), 10.193 (3), 30.479 (7)	21.441 (4), 5.2059 (9), 17.465 (4)
α, β, γ°	90, 90, 90	90, 102.673 (10), 90
V (Å^3)	1484.0 (7)	1901.9 (7)
Z	4	4
Density (calculated)	1.358 *Mg/m*^{3}	1.995 *Mg/m*^{3}
F(000)	640	1088

Radiation type	Mo *K*α	Mo *K*α
Wavelength (λ)	0.71073 Å	0.71073 Å
μ (mm^{-1})	0.102	3.435
Crystal size (*mm*)	0.40 × 0.22 × 0.16	0.43 × 0.20 × 0.14
**Data collection**		
Diffractometer	Bruker APEXII CCD diffractometer	Bruker APEXII CCD diffractometer
Absorption correction	multi-scan (SADABS; Bruker, 2007)	multi-scan (SADABS; Bruker, 2007)
No. of measured, independent and observed [$I > 2\sigma(I)$] reflections	6629, 2753, 1398	11274, 4128, 2516
R_{int}	0.082	0.049
Theta range for data collection (°)	2.673-25.498	2.921 -27.000
Index ranges	-5≤ h ≤4, -10≤ k ≤ 12, -32≤ l ≤ 36	-25≤ h ≤27, -6≤ k ≤6, -16≤ l ≤ 22
$(\sin \theta/\lambda)_{max}$ (Å^{-1})	0.606	0.639
**Refinement**		
$R[F^2 > 2\sigma(F^2)]$, $wR(F^2)$, S	0.091, 0.189, 1.09	0.044, 0.103, 1.01
No. of reflections	2753	4128
No. of parameters	196	186
H-atom treatment	H atoms treated by a mixture of independent and constrained refinement	H-atom parameters constrained
$\Delta\rho_{max}$, $\Delta\rho_{min}$ (e Å^{-3})	0.27, −0.26	0.87, −0.75

Table S2: Comparison of XRD and DFT values of selected bond lengths and bond angles of **HMBA** using m06/6-311g(d,p).

Bond lengths			Bond angles		
	DFT	EXP		DFT	EXP
O1-C1	1.202	1.207	O1-C1-O2	122.3	122.7
O2-C1	1.347	1.312	O1-C1-C2	125.1	123.9
O3-C10	1.242	1.303	O2-C1-C2	112.6	113.4
O4-C11	1.346	1.357	O3-C10-C9	122.8	123.6
O4-C15	1.403	1.443	O3-C10-C11	121.4	119.6
N1-C5	1.402	1.433	C11-O4-C15	117.4	116.8
N1-C8	1.33	1.278	O4-C11-C10	113	112.9
C1-C2	1.476	1.49	O4-C11-C12	126	127.1
C2-C3	1.393	1.374	C5-N1-C8	126.4	125.4
C2-C7	1.393	1.383	N1-C5-C4	122.3	122.3

C3-C4	1.381	1.375	N1-C5-C6	117.7	117.2
C4-C5	1.395	1.371	N1-C8-C9	126.5	121.9
C5-C6	1.397	1.37	C1-C2-C3	122.4	122.6
C6-C7	1.38	1.382	C1-C2-C7	118.3	119.7
C8-C9	1.384	1.427	C3-C2-C7	119.3	117.7
C9-C10	1.455	1.403	C2-C3-C4	120.5	122.7
C9-C14	1.432	1.407	C2-C7-C6	120.7	120.4
C10-C11	1.46	1.451	C3-C4-C5	119.8	118.4
C11-C12	1.363	1.367	C4-C5-C6	120	120.5
C12-C13	1.427	1.399	C5-C6-C7	119.6	120.1
C13-C14	1.352	1.369	C8-C9-C10	120.6	119.6
O5-C16	1.404	1.419	C8-C9-C14	118.7	118.8

EXP=experiment, DFT=density functional theory

Table S2: Continued…

Bond angles		
	DFT	EXP
C10-C9-C14	120.8	121.6
C9-C10-C11	115.8	116.8
C9-C14-C13	120.5	120.2
C10-C11-C12	121	120.1
C11-C12-C13	121.6	121.7
C12-C13-C14	120.3	119.7

EXP=experiment, DFT=density functional theory

Table S3: Compassion of XD and DFT values of selected bond lengths and bond angles of **DHB** using m06/6-311g(d,p).

**Bond lengths**			**Bond angles**		
	**DFT**	**EXP**		**DFT**	**EXP**
I1-C13	2.13	2.094	I1-C13-C28	119.5	120.6
I2-C11	2.12	2.082	I1-C13-C14	120.2	119.7
O1-C1	1.21	1.196	I2-C11-C10	119.2	119.1
O2-C1	1.347	1.311	I2-C11-C12	119.6	119.3
O3-C10	1.325	1.318	O1-C1-O2	122.4	122.8
N1-C5	1.401	1.409	O1-C1-C2	124.8	124.3
N1-C8	1.286	1.272	O2-C1-C2	112.8	112.8
C1-C2	1.479	1.474	O3-C10-C9	122.2	121.3
C2-C3	1.396	1.39	O3-C10-C11	119.6	120.4
C2-C7	1.397	1.347	C5-N1-C8	120.8	122.9
C3-C4	1.386	1.389	N1-C5-C4	122.4	122.7
C4-C5	1.401	1.39	N1-C5-C6	117.9	117.3
C5-C6	1.4	1.39	N1-C8-C9	122.5	121.9
C6-C7	1.383	1.389	C1-C2-C3	122.3	121.5
C8-C9	1.448	1.428	C1-C2-C7	117.9	119.7
C9-C10	1.419	1.416	C3-C2-C7	119.8	116.9
C9-C14	1.402	1.384	C2-C3-C4	120.1	120
C10-C11	1.404	1.398	C2-C7-C6	120.2	120.5

C11-C12	1.386	1.366	C3-C4-C5	120	120
C12-C13	1.397	1.388	C4-C5-C6	119.7	120
C13-C14	1.38	1.373	C5-C6-C7	120	120
S1-O4	1.497	1.508	C8-C9-C10	121.1	120.9
S1-C15	1.824	1.754	C8-C9-C14	118.8	120.2
S1-C16	1.82	1.758	C10-C9-C14	120.1	118.9

EXP=experiment, DFT=density functional theory

Table S3: Continued…

**Bond angles**		
	**DFT**	**EXP**
C9-C10-C11	118.1	118.3
C9-C15-C13	120.3	121.6
C10-C11-C12	121.3	121.5
C11-C12-C13	119.9	119.9
C12-C13-C14	120.3	119.6
O4-S1-C15	106.8	108.1
O4-S1-C16	107.1	105.7
C15-S1-C16	94.5	98.2

EXP=experiment, DFT=density functional theory

Table S4: Enrichment ratio of pair of chemical species of the **HMBA.** The enrichment ratio is not calculated for pair of chemical species for which random contact is less than 0.9%.

**Contact %**	**Atom**	**H**	**C**	**N**	**O**
	**H**	42.2	15.4	0	31.1
	**C**	15.4	7.2	2.3	1.7
	**N**	0	2.3	0	0
	**O**	31.1	1.7	0	0.2
**Surface% S_{XY}**		65.45	16.9	1.15	16.6
**Random Contacts % R_{XY}**	**Atom**	**H**	**C**	**N**	**O**
	**H**	42.84	-	-	-
	**C**	22.12	2.86	-	-
	**N**	1.51	0.39	0.01	
	**O**	21.73	5.61	0.38	2.76
**Enrichment ratio E_{CH}**	**Atom**	**H**	**C**	**N**	**O**
	**H**	0.99	-	**-**	**-**
	**C**	0.70	2.52	-	-
	**N**	0.00	-	-	-
	**O**	1.43	0.30	-	0.07

Table S5: Enrichment ratio of pair of chemical species of the **DHBA.** The enrichment ratio is not calculated for pair of chemical species for which random contact is less than 0.9%.

**Contact %**	**Atom**	**H**	**C**	**N**	**O**	**S**	**I**
	**H**	22.1	13.6	1.1	24.3	3.9	17.3
	**C**	13.6	2.4	0	3.1	0	4

	**N**	1.1	0	0	0.1	0	0
	**O**	24.3	3.1	0	0.3	0	0
	**S**	3.9	0	0	0	0	0
	**I**	17.3	4	0	0	0	7.3
**Surface% S_{XY}**		52.2	12.75	0.55	14.05	1.95	17.95
**Random Contacts % R_{XY}**	**Atom**	**H**	**C**	**N**	**O**	**S**	**I**
	**H**	27.25					
	**C**	13.31	1.63				
	**N**	0.57	0.14	0.00			
	**O**	14.67	3.58	0.15	1.97		
	**S**	2.04	0.50	0.02	0.55	0.04	
	**I**	18.74	4.58	0.20	5.04	0.70	3.22
**Enrichment ratio E_{CH}**	**Atom**	**H**	**C**	**N**	**O**	**S**	**I**
	**H**	0.81	-	-	-	-	-
	**C**	1.02	1.48	-	-	-	-
	**N**	-	-	-	-	-	-
	**O**	1.66	0.87	-	0.15	-	-
	**S**	1.92	-	-	-	-	-
	**I**	0.92	0.87	-	0	-	2.27

Table S6: Natural bond orbital (NBO) analysis of **HMBA** compound.

Donor(i)	Type	Acceptor(j)	Type	E(2)[a] [kJ/mol]	E(J)E(i)[b](a.u)	F(I,j)[c](a.u)
C8-H20	σ	C9-C10	σ*	5.15	1.06	0.066
C3-H11	σ	C4-C5	σ*	4.39	1.09	0.062
C13-H27	σ	C11-C12	σ*	3.78	1.13	0.058
C9-C10	σ	O3-C10	σ*	2.02	1.25	0.045
C15-H31	σ	O4-C11	σ*	0.55	0.92	0.02
C4-C5	π	C2-C3	π*	26.32	0.31	0.08
C6-C7	π	C4-C5	π*	25.27	0.29	0.077
C2-C3	π	C6-C7	π*	23.4	0.3	0.077
C2-C3	π	O1-C1	π*	22.93	0.29	0.075
C11-C12	π	O3-C10	π*	22.75	0.3	0.077
C2-C3	π	C4-C5	π*	19.41	0.29	0.067
C6-C7	π	C2-C3	π*	19.2	0.3	0.068
C4-C5	π	C6-C7	π*	16.96	0.31	0.066
C13-C14	π	C11-C12	π*	16.48	0.31	0.066
N1-C8	π	C4-C5	π*	15.95	0.4	0.078
C11-C12	π	C13-C14	π*	15.18	0.33	0.064
C4-C5	π	N1-C8	π*	7.97	0.24	0.04
C2-C3	π	C2-C3	π*	0.61	0.3	0.012
O2	LP(2)	O1-C1	π*	47.09	0.37	0.121
O1	LP(2)	O2-C1	σ*	34.41	0.65	0.135
O4	LP(2)	C11-C12	π*	34.12	0.37	0.103
O3	LP(2)	C10-C11	σ*	18.71	0.78	0.109
O1	LP(2)	C1-C2	σ*	18.6	0.74	0.107
O3	LP(2)	C9-C10	σ*	13.65	0.79	0.094

O3	LP(2)	N1-H7	σ*	7.91	0.68	0.067
O4	LP(1)	C11-C12	σ*	7.32	1.18	0.083

Table S7: Natural bond orbital (NBO) analysis of **DHBA** compound.

**Donor(i)**	**Type**	**Acceptor(j)**	**Type**	**E(2)[a] [kJ/mol]**	**E(J)E(i)[b](a.u)**	**F(I,j)[c](a.u)**
C9-C10	σ	C10-C11	σ*	6.42	1.26	0.08
O3-H7	σ	C10-C11	σ*	5.83	1.3	0.078
C11-C12	σ	C12-C13	σ*	5.51	1.31	0.076
I2-C11	σ	C12-C13	σ*	5.03	1.07	0.065
C6-C7	σ	C2-C7	σ*	3.9	1.3	0.064
C11-C12	σ	O3-C10	σ*	3.22	1.15	0.054
N1-C8	σ	C5-C6	σ*	2.14	1.48	0.05
C5-C6	σ	N1-C5	σ*	2.01	1.15	0.043
O3-H7	σ	N1-C5	σ*	0.52	1.21	0.022
C10-C11	π	C12-C13	π*	30.68	0.3	0.086
C12-C13	π	C9-C14	π*	28.03	0.3	0.084
C9-C14	π	C10-C11	π*	26.58	0.28	0.078
C4-C5	π	C2-C3	π*	25.93	0.3	0.079
C6-C7	π	C4-C5	π*	24.77	0.29	0.077
C9-C14	π	N1-C8	π*	24.08	0.29	0.078
C2-C3	π	C6-C7	π*	22.65	0.3	0.076
C2-C3	π	O1-C1	π*	22.31	0.29	0.074
C6-C7	π	C2-C3	π*	19.88	0.3	0.069
C2-C3	π	C4-C5	π*	19.52	0.29	0.068
C9-C14	π	C12-C13	π*	18.3	0.29	0.065
C4-C6	π	C6-C7	π*	17.44	0.31	0.067
C12-C13	π	C10-C11	π*	16.07	0.29	0.062
C10-C11	π	C9-C14	π*	15.69	0.31	0.063
N1-C8	π	C4-C5	π*	10.09	0.4	0.061
C4-C5	π	N1-C8	π*	8.22	0.28	0.045
N1-C8	π	C9-C14	π*	7.06	0.38	0.051
C9-C14	π	C9-C14	π*	0.67	0.29	0.013
O2	LP(2)	O1-C1	π*	47.53	0.37	0.121
O3	LP(2)	C10-C11	π*	42.41	0.34	0.115
O1	LP(2)	O2-C1	σ*	34.35	0.65	0.135
N1	LP(1)	O3-H7	σ*	21.77	0.79	0.12
O1	LP(2)	C1-C2	σ*	18.72	0.73	0.107
N1	LP(1)	C8-H21	σ*	11.86	0.74	0.086
O3	LP(1)	C9-C10	σ*	8.12	1.13	0.086
O2	LP(1)	O1-C1	σ*	6.1	1.26	0.078
N1	LP(1)	C4-C5	σ*	5.78	0.95	0.068
N1	LP(1)	C4-C5	π*	5.65	0.41	0.046
I2	LP(2)	C11-C12	σ*	2.14	0.84	0.038
I2	LP(1)	C10-C11	σ*	0.54	1.15	0.023

Com= compounds; LP= lone pair, (j) acceptor, (i) donor, $E^{(2)}$ means energy of hyper conjugative interaction (stabilization energy), F(i, j) is the Fock matrix element between i and j NBO orbitals, E(j)-E(i) is the energy difference between donor and acceptor i and j NBO orbitals.

Table S8: Dipole polarizability and major contributing tensors (*a.u.*) of **HMBA** and **DHBA** compounds.

**Dipole Moment**	**HMBA**	**DHBA**	**Polarizability**	**HMBA**	**DHBA**
μ_x	-2.74	0.84	α_{xx}	528.79	436.95
μ_y	-5.55	5.68	α_{yy}	278.96	262.83
μ_z	-2.06	-0.43	α_{zz}	132.68	166.58
μ_{total}	6.53	5.76	α_{total}	313.48	288.79

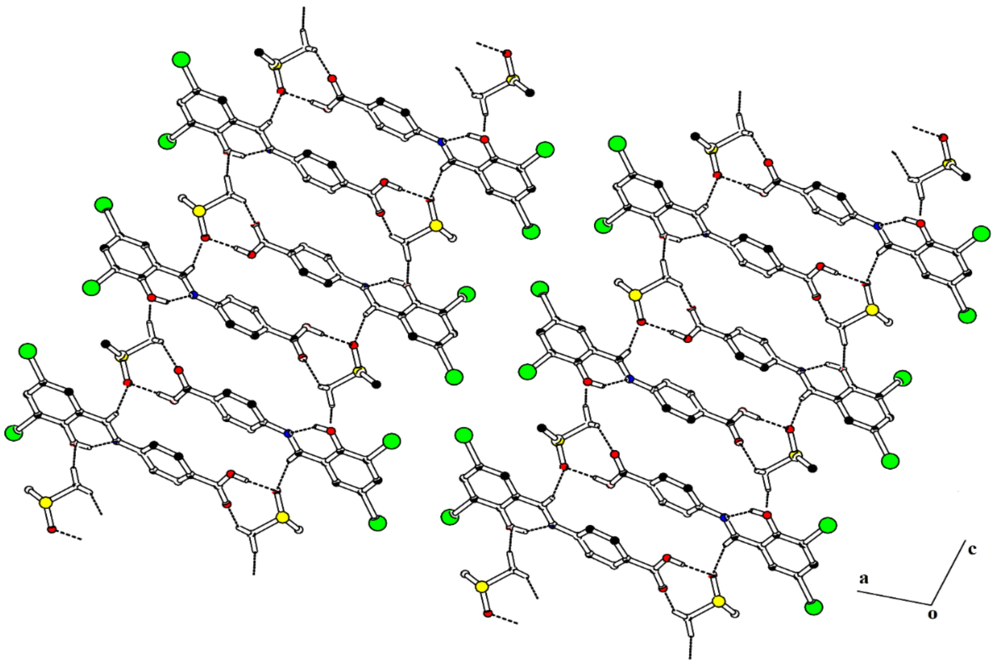

Figure S1. Intermolecular and intramolecular H-bonding in packed crystal structure of **DHBA**. Selected H-atoms are shown for clarity.

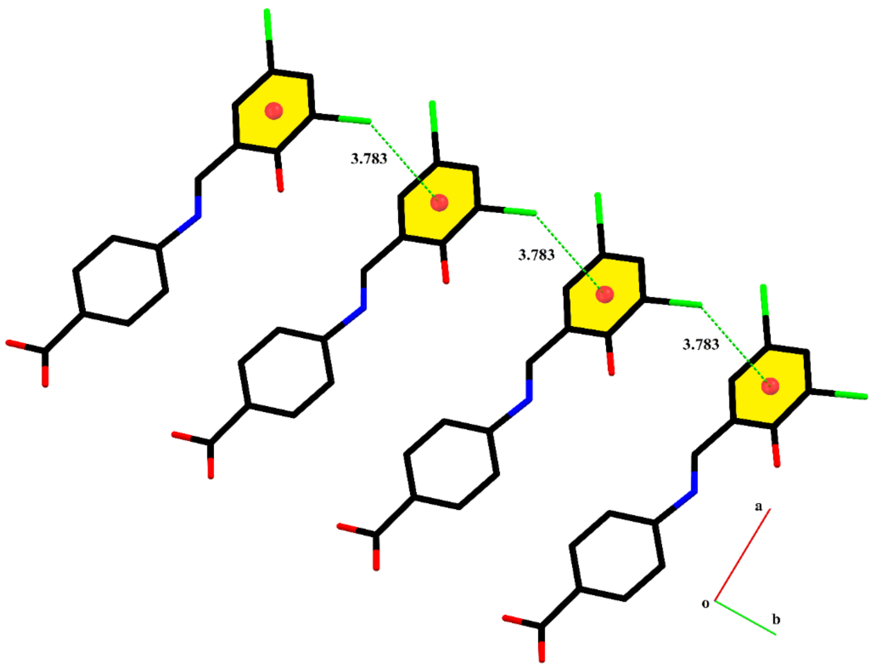

Figure S2. Graphical illustration of C-I···π interactions in **DHBA**. H-atoms and solvent molecules are not represented for simplicity.

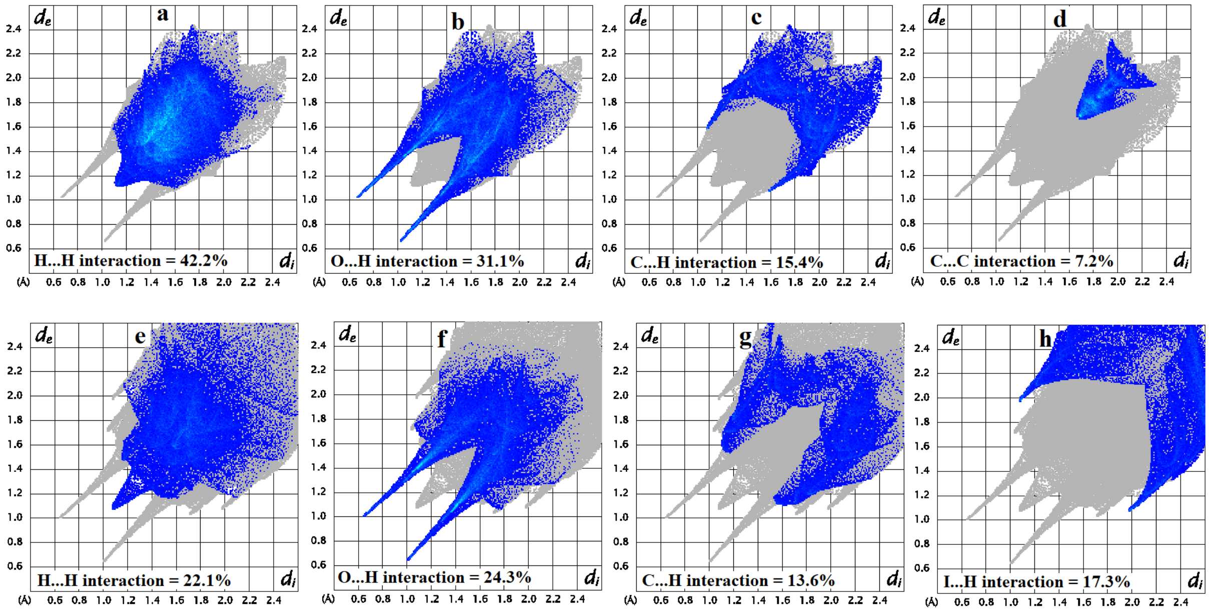

Figure S3. Important 2 D finger print plots for **(a-d) HMBA**, **(e-h) DHBA**.

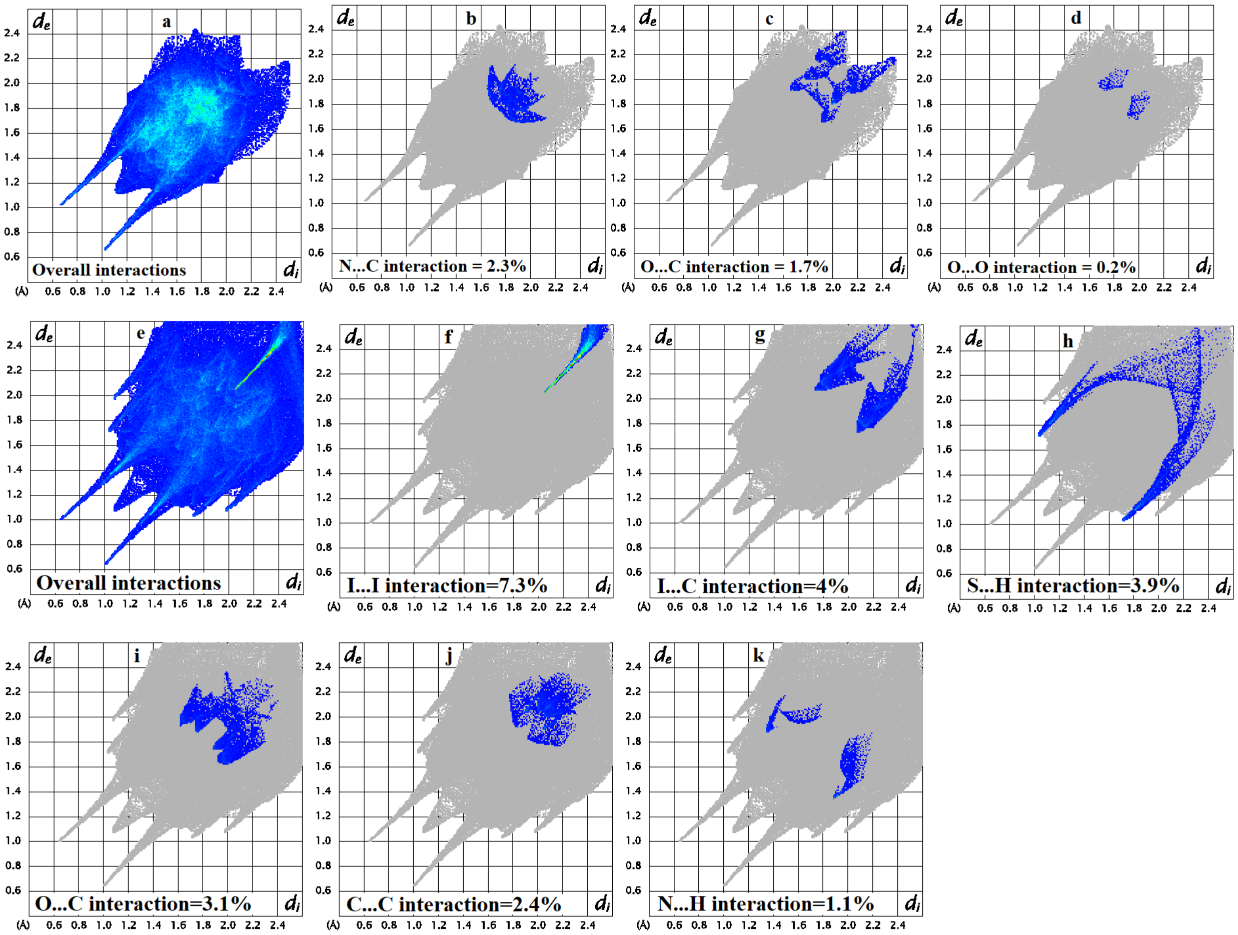

Figure S4. Remaining 2D finger print plots (a-d) for **HMBA** and **(e-k)** for **DHBA**. The 2d plot of the interatomic contacts with contribution less than 1% are not shown.

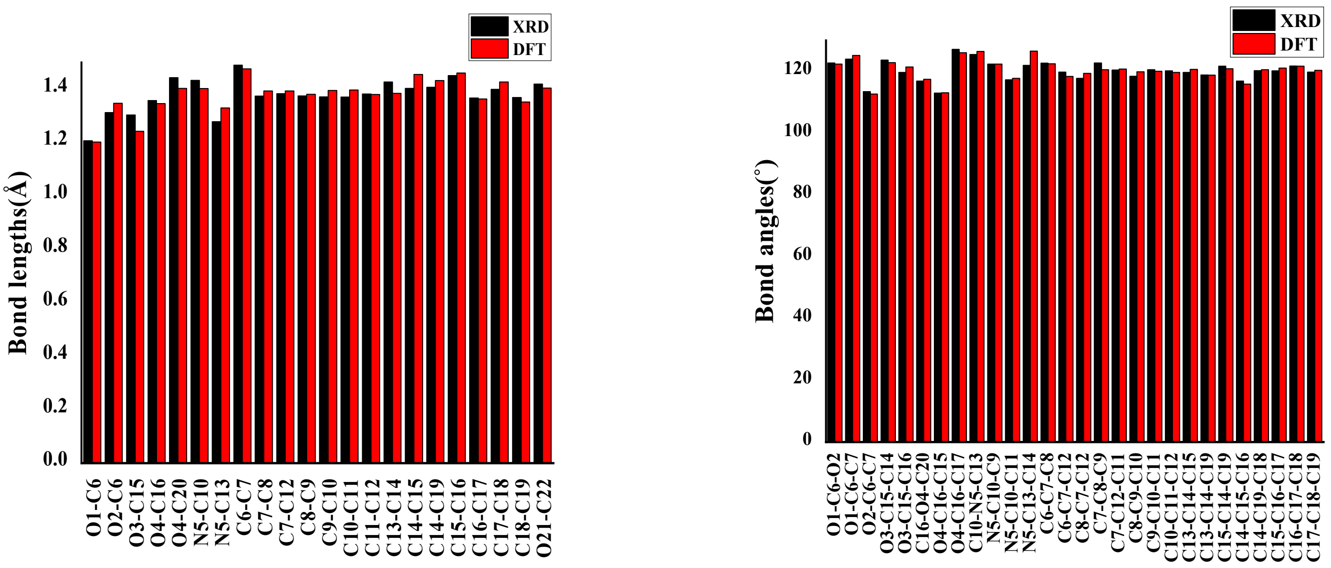

HMBA **HMBA**

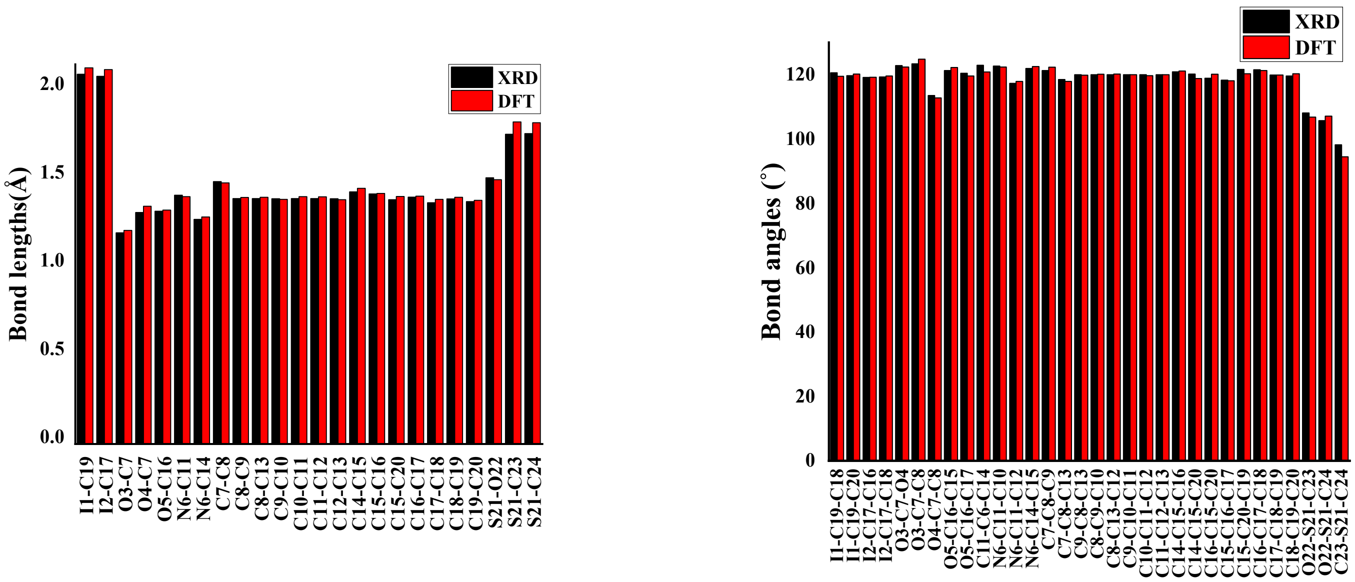

DHBA **DHBA**

Figure S5: Bond lengths (Å) and bond angles (°) for **HMBA** and **DHBA**.

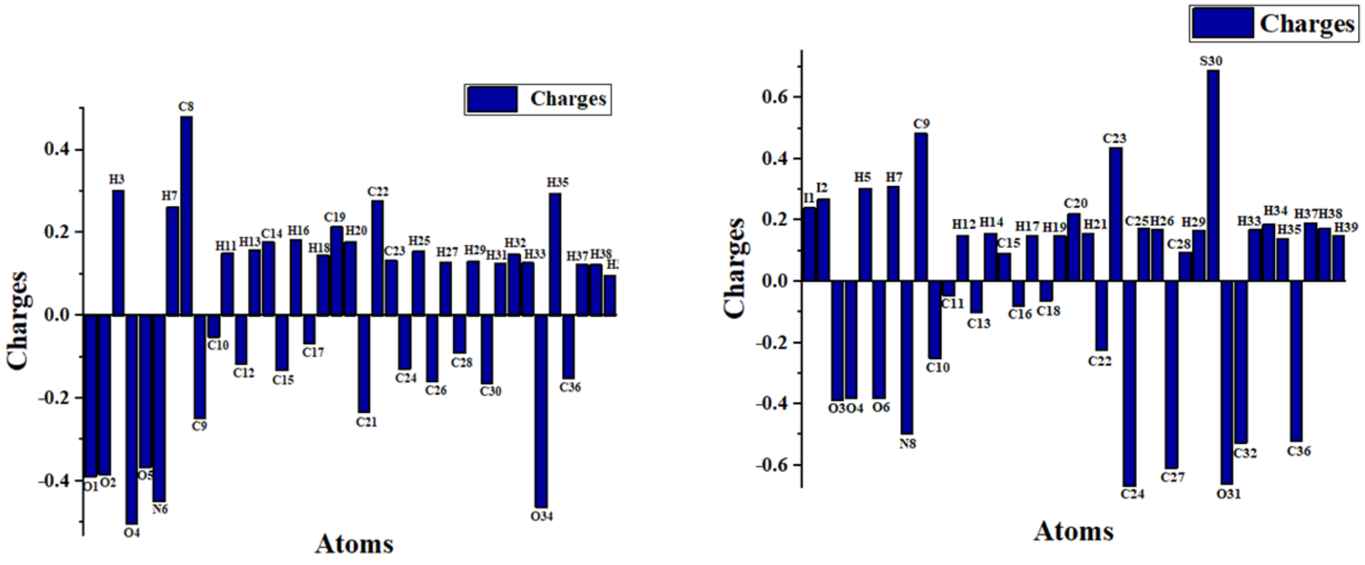

HMBA **DHBA**

Figure S6. The natural charge distribution of **HMBA** and **DHBA** compounds.

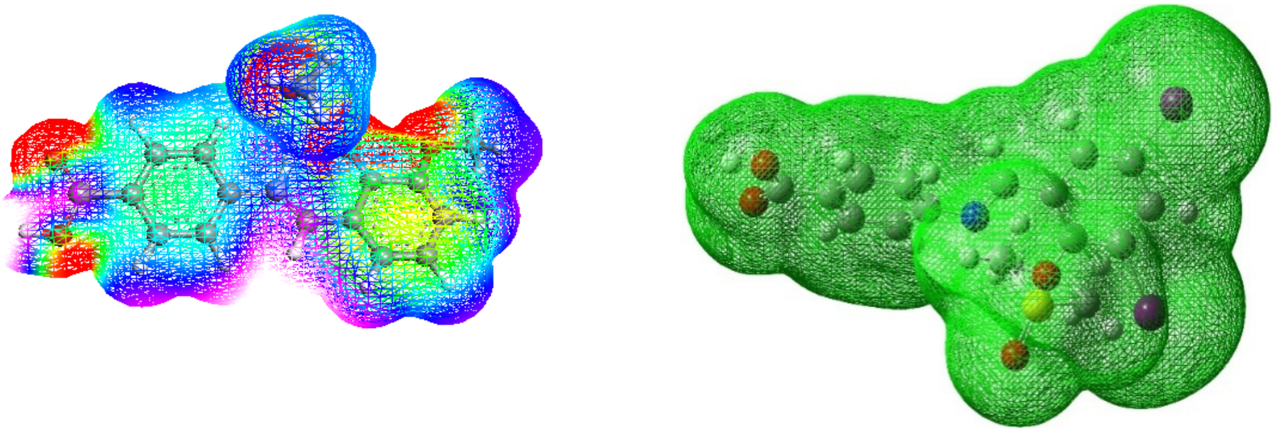

HMBA **DHBA**

Figure S7. MEPs and color scheme of the investigated compounds: **HMBA** and **DHBA**.

checkCIF/PLATON report of HMBA

Structure factors have been supplied for datablock(s) HMBA

THIS REPORT IS FOR GUIDANCE ONLY. IF USED AS PART OF A REVIEW PROCEDURE FOR PUBLICATION, IT SHOULD NOT REPLACE THE EXPERTISE OF AN EXPERIENCED CRYSTALLOGRAPHIC REFEREE.

No syntax errors found. CIF dictionary Interpreting this report

Datablock: HMBA

Bond precision:	C-C = 0.0097 A	Wavelength=0.71073	
Cell:	a=4.7768(11)	b=10.193(3)	c=30.479(7)
	alpha=90	beta=90	gamma=90
Temperature:	296 K		

	Calculated	Reported
Volume	1484.0(7)	1484.0(7)
Space group	P 21 21 21	P 21 21 21
Hall group	P 2ac 2ab	P 2ac 2ab
Moiety formula	C15 H13 N O4, C H4 O	C15 H13 N O4, C H4 O
Sum formula	C16 H17 N O5	C16 H17 N O5
Mr	303.31	303.30
Dx,g cm-3	1.358	1.358
Z	4	4
Mu (mm-1)	0.102	0.102
F000	640.0	640.0
F000'	640.36	
h,k,lmax	5,12,36	5,12,36
Nref	2766[1659]	2753
Tmin,Tmax	0.973,0.984	0.915,0.975
Tmin'	0.960	

Correction method= # Reported T Limits: Tmin=0.915 Tmax=0.975 AbsCorr = MULTI-SCAN

Data completeness= 1.66/1.00 Theta(max)= 25.498

R(reflections)= 0.0910(1398) wR2(reflections)= 0.1885(2753)

S = 1.087 Npar= 196

The following ALERTS were generated. Each ALERT has the format

test-name_ALERT_alert-type_alert-level.

Click on the hyperlinks for more details of the test.

Alert level C

STRVA01_ALERT_4_C Flack test results are meaningless. From the CIF: _refine_ls_abs_structure_Flack -0.100
From the CIF: _refine_ls_abs_structure_Flack_su 1.000

PLAT340_ALERT_3_C	Low Bond Precision on C-C Bonds	0.00971	Ang.
PLAT906_ALERT_3_C	Large K Value in the Analysis of Variance	2.744	Check
PLAT906_ALERT_3_C	Large K Value in the Analysis of Variance	2.746	Check

Alert level G

PLAT002_ALERT_2_G	Number of Distance or Angle Restraints on AtSite	6	Note
PLAT003_ALERT_2_G	Number of Uiso or Uij Restrained non-H Atoms ...	14	Report
PLAT007_ALERT_5_G	Number of Unrefined Donor-H Atoms	1	Report
PLAT032_ALERT_4_G	Std. Uncertainty on Flack Parameter Value High .	1.000	Report
PLAT172_ALERT_4_G	The CIF-Embedded .res File Contains DFIX Records	1	Report
PLAT178_ALERT_4_G	The CIF-Embedded .res File Contains SIMU Records	2	Report
PLAT309_ALERT_2_G	Single Bonded Oxygen (C-O > 1.3 Ang)	O3	Check
PLAT910_ALERT_3_G	Missing # of FCF Reflection(s) Below Theta(Min).	3	Note
PLAT916_ALERT_2_G	Hooft y and Flack x Parameter Values Differ by .	0.50	Check
PLAT933_ALERT_2_G	Number of HKL-OMIT Records in Embedded .res File	2	Note
PLAT941_ALERT_3_G	Average HKL Measurement Multiplicity	4.0	Low
PLAT967_ALERT_5_G	Note: Two-Theta Cutoff Value in Embedded .res ..	51.0	Degree
PLAT978_ALERT_2_G	Number C-C Bonds with Positive Residual Density.	1	Info

0 **ALERT level A** = Most likely a serious problem - resolve or explain

0 **ALERT level B** = A potentially serious problem, consider carefully

4 **ALERT level C** = Check. Ensure it is not caused by an omission or oversight

13 **ALERT level G** = General information/check it is not something unexpected

0	ALERT	type	1	CIF construction/syntax error, inconsistent or missing data
6	ALERT	type	2	Indicator that the structure model may be wrong or deficient
5	ALERT	type	3	Indicator that the structure quality may be low
4	ALERT	type	4	Improvement, methodology, query or suggestion
2	ALERT	type	5	Informative message, check

It is advisable to attempt to resolve as many as possible of the alerts in all categories. Often the minor alerts point to easily fixed oversights, errors and omissions in your CIF or refinement strategy, so attention to these fine details can be worthwhile. In order to resolve some of the more serious problems it may be necessary to carry out additional measurements or structure refinements. However, the purpose of your study may justify the reported deviations and the more serious of these should normally be commented upon in the discussion or experimental section of a paper or in the "special_details" fields of the CIF. checkCIF was carefully designed to identify outliers and unusual parameters, but every test has its limitations and alerts that are not important in a particular case may appear. Conversely, the absence of alerts does not guarantee there are no aspects of the results needing attention. It is up to the individual to critically assess their own results and, if necessary, seek expert advice.

Publication of your CIF in IUCr journals

A basic structural check has been run on your CIF. These basic checks will be run on all CIFs submitted for publication in IUCr journals (*Acta Crystallographica*, *Journal of Applied Crystallography*, *Journal of Synchrotron Radiation*); however, if you intend to submit to *Acta Crystallographica Section C* or *E* or *IUCrData*, you should make sure that full publication checks are run on the final version of your CIF prior to submission.

Publication of your CIF in other journals

Please refer to the *Notes for Authors* of the relevant journal for any special instructions relating to CIF submission.

PLATON version of 20/01/2022; check.def file version of 19/01/2022

Datablock new4abovan_0m - ellipsoid plot

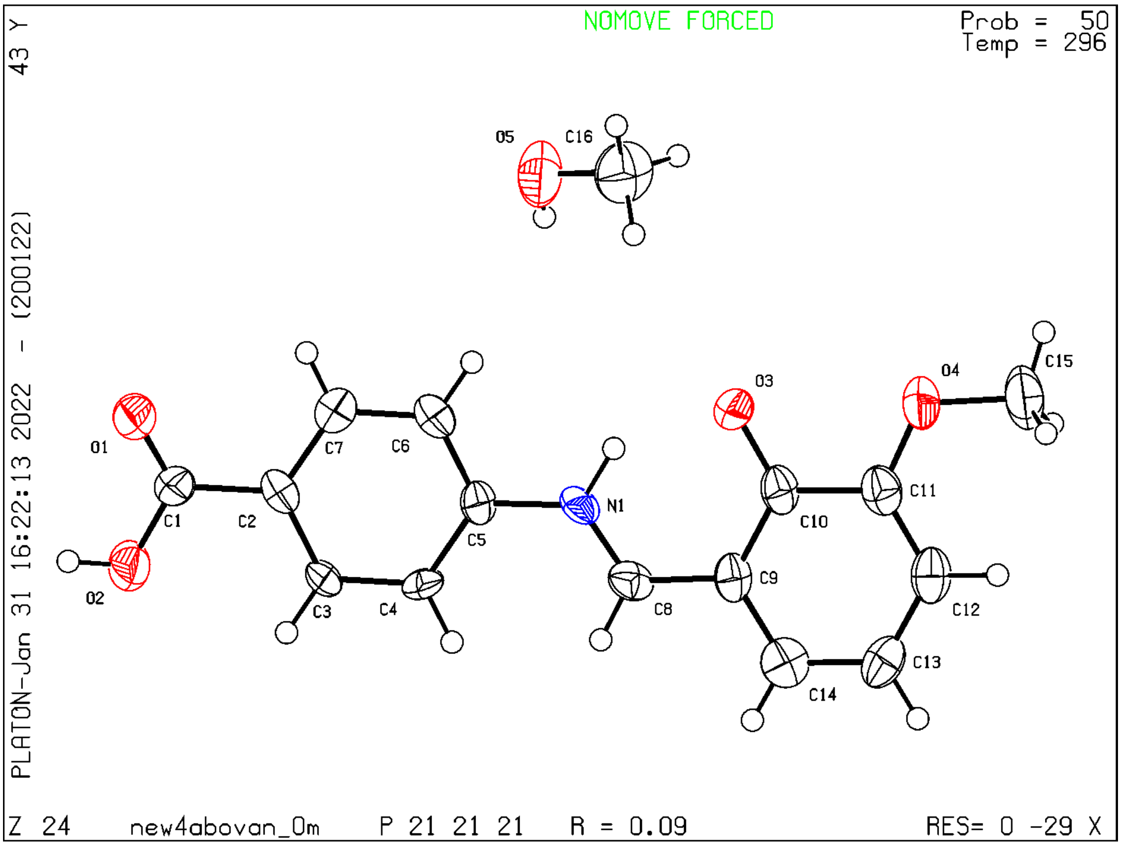

checkCIF/PLATON report of DHBA

Structure factors have been supplied for datablock(s) DHBA

THIS REPORT IS FOR GUIDANCE ONLY. IF USED AS PART OF A REVIEW PROCEDURE FOR PUBLICATION, IT SHOULD NOT REPLACE THE EXPERTISE OF AN EXPERIENCED CRYSTALLOGRAPHIC REFEREE.

No syntax errors found. CIF dictionary Interpreting this report

Datablock: DHBA

Bond precision: C-C = 0.0071 A Wavelength=0.71073

Cell:	a=21.441(4)	b=5.2059(9)	c=17.465(4)
	alpha=90	beta=102.673(10)	gamma=90
Temperature:	296 K		

	Calculated	Reported
Volume	1902.0(7)	1901.9(7)
Space group	P 21/c	P 21/c
Hall group	-P 2ybc	-P 2ybc

Moiety formula C14 H9 I2 N O3, C2 H6 O S C16 H15 I2 N O4 S Sum formula C16 H15 I2 N O4 S C16 H15 I2 N O4 S Mr 571.15 571.15

	Calculated	Reported
Dx,g cm-3	1.995	1.995
Z	4	4
Mu (mm-1)	3.435	3.435
F000	1088.0	1088.0
F000’	1085.14	
h,k,lmax	27,6,22	27,6,22
Nref	4153	4128
Tmin,Tmax	0.443,0.618	0.945,0.985
Tmin’	0.226	

Correction method= # Reported T Limits: Tmin=0.945 Tmax=0.985 AbsCorr = MULTI-SCAN

Data completeness= 0.994 Theta(max)= 27.000

R(reflections)= 0.0439(2516) wR2(reflections)= 0.1028(4128)

S = 1.008 Npar= 186

The following ALERTS were generated. Each ALERT has the format

test-name_ALERT_alert-type_alert-level.

Click on the hyperlinks for more details of the test.

Alert level C

PLAT244_ALERT_4_C Low 'Solvent' Ueq as Compared to Neighbors of S1 Check PLAT910_ALERT_3_C Missing # of FCF Reflection(s) Below Theta(Min). 6 Note
PLAT911_ALERT_3_C Missing FCF Refl Between Thmin & STh/L= 0.600 16 Report

Alert level G

PLAT007_ALERT_5_G Number of Unrefined Donor-H Atoms .... 2 Report
PLAT042_ALERT_1_G Calc. and Reported Moiety Formula Strings Differ Please Check
PLAT171_ALERT_4_G The CIF-Embedded .res File Contains EADP Records 1 Report

PLAT301_ALERT_3_G	Main	Residue	Disorder (Resd			1	) Note	30%		
PLAT410_ALERT_2_G	Short Intra H...H			Contact	H4A	..H8	.	2.04	Ang.	
						x,y,z	=	1_555	Check	
PLAT410_ALERT_2_G	Short Intra H...H			Contact	H8	..H4B	.	2.04	Ang.	
						x,y,z	=	1_555	Check	
PLAT480_ALERT_4_G	Long	H...A	H-Bond	Reported	H2	..S1	.	2.95	Ang.	
PLAT480_ALERT_4_G	Long	H...A	H-Bond	Reported	H6B	..O1	.	2.61	Ang.	
PLAT480_ALERT_4_G	Long	H...A	H-Bond	Reported	H12	..I2	.	3.19	Ang.	
PLAT480_ALERT_4_G	Long	H...A	H-Bond	Reported	H14	..S1	.	2.89	Ang.	

PLAT912_ALERT_4_G Missing # of FCF Reflections Above STh/L= 0.600 4 Note
PLAT933_ALERT_2_G Number of HKL-OMIT Records in Embedded .res File 4 Note
PLAT941_ALERT_3_G Average HKL Measurement Multiplicity .... 2.7 Low
PLAT967_ALERT_5_G Note: Two-Theta Cutoff Value in Embedded .res .. 54.0 Degree
PLAT978_ALERT_2_G Number C-C Bonds with Positive Residual Density. 6 Info

0 **ALERT level A** = Most likely a serious problem - resolve or explain

0 **ALERT level B** = A potentially serious problem, consider carefully

3 **ALERT level C** = Check. Ensure it is not caused by an omission or oversight

15 **ALERT level G** = General information/check it is not something unexpected

1	ALERT	type	1	CIF construction/syntax error, inconsistent or missing data
4	ALERT	type	2	Indicator that the structure model may be wrong or deficient
4	ALERT	type	3	Indicator that the structure quality may be low
7	ALERT	type	4	Improvement, methodology, query or suggestion
2	ALERT	type	5	Informative message, check

It is advisable to attempt to resolve as many as possible of the alerts in all categories. Often the minor alerts point to easily fixed oversights, errors and omissions in your CIF or refinement strategy, so attention to these fine details can be worthwhile. In order to resolve some of the more serious problems it may be necessary to carry out additional measurements or structure refinements. However, the purpose of your study may justify the reported deviations and the more serious of these should normally be commented upon in the discussion or experimental section of a paper or in the "special_details" fields of the CIF. checkCIF was carefully designed to identify outliers and unusual parameters, but every test has its limitations and alerts that are not important in a particular case may appear. Conversely, the absence of alerts does not guarantee there are no aspects of the results needing attention. It is up to the individual to critically assess their own results and, if necessary, seek expert advice.

Publication of your CIF in IUCr journals

A basic structural check has been run on your CIF. These basic checks will be run on all CIFs submitted for publication in IUCr journals (*Acta Crystallographica*, *Journal of Applied Crystallography*, *Journal of Synchrotron Radiation*); however, if you intend to submit to *Acta Crystallographica Section C* or *E* or *IUCrData*, you should make sure that full publication checks are run on the final version of your CIF prior to submission.

Publication of your CIF in other journals

Please refer to the *Notes for Authors* of the relevant journal for any special instructions relating to CIF submission.

PLATON version of 20/01/2022; check.def file version of 19/01/2022

Datablock 4aba35diiodo - ellipsoid plot

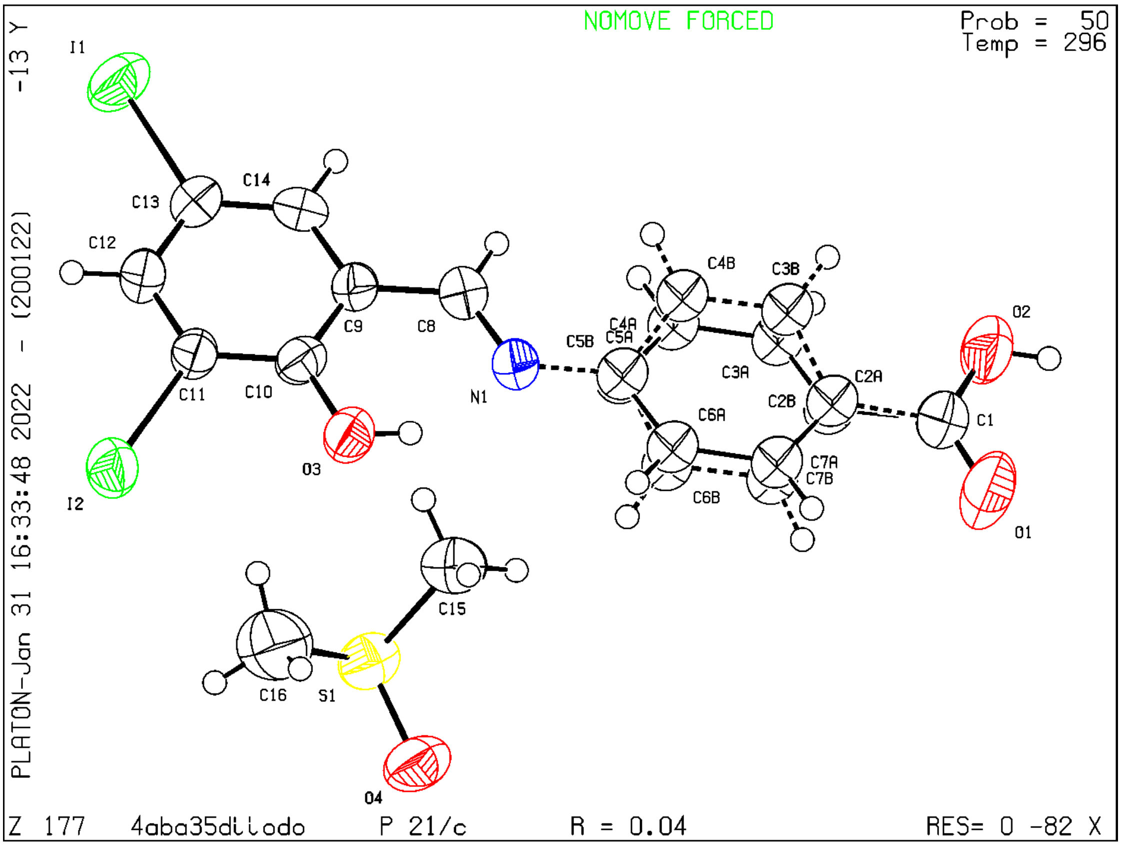

CIF of HMBA without hkl data

data_global

#==

PROCESSING SUMMARY (IUCr Office Use only)

_publ_contact_author

;

Muhammad Nawaz Tahir

University of Sargodha

Department of Physics

Sargodha

Pakistan

;

_publ_contact_author_phone '0092 48 92 30 914'

_publ_contact_author_fax '0092 48 32 22 121'

_publ_contact_author_email 'dmntahir_uos@yahoo.com'

_publ_requested_journal ''

_journal_date_recd_electronic ?

_journal_date_to_coeditor ?

_journal_date_from_coeditor ?

_journal_date_accepted ?

_journal_date_printers_first ?

_journal_date_printers_final ?

_journal_date_proofs_out ?

_journal_date_proofs_in ?

_journal_coeditor_name ?

_journal_coeditor_code ?

_journal_paper_category ?

_journal_coeditor_notes

;

;

```
_journal_techeditor_code      ?
_iucr_compatibility_tag          ?
_journal_techeditor_notes
;
;

_journal_coden_ASTM               ?
_journal_name_full                ?
_journal_year                     ?
_journal_volume                   ?
_journal_issue                    ?
_journal_page_first               ?
_journal_page_last                ?

_journal_suppl_publ_number        ?
_journal_suppl_publ_pages         ?
#==============================================================

 loop_
_publ_author_name
_publ_author_address

'Muhammad Nawaz Tahir'
;
```

Department of Physics

University of Sargodha

Sargodha

Pakistan

;

'Muhammad Ashfaq'

;

Department of Physics

University of Sargodha

Sargodha

Pakistan

;

'Akbar Ali'

;

Department of Chemistry,

Government College University,

Faisalabad,

Pakistan

;

'Muhammad Khalid'

;

Department of Chemistry,

Khwaja Fareed University of Engineering and Information Technology,

Rahim Yar Khan,64200,

Pakistan

;

_audit_creation_date ?

_audit_creation_method ?

#==

TEXT

_publ_section_title

;

2-{(E)-[(4-carboxyphenyl)iminio]methyl}-6-methoxyphenolate methanol (1/1)

;

_publ_section_abstract

;

;

_publ_section_comment

;

;

_publ_section_exptl_refinement

;

;

_publ_section_exptl_prep

;

;

_publ_section_related_literature

;

;

_publ_section_references

;

Bruker (2005). <i>SADABS</i>. Bruker AXS Inc., Madison, Wisconsin, USA.

Bruker (2007). <i>APEX2</i> and <i>SAINT</i>. Bruker AXS Inc., Madison, Wisconsin, USA.

Farrugia, L. J. (1997). <i>J. Appl. Cryst.</i> 30, 565.

Farrugia, L. J. (1999). <i>J. Appl. Cryst.</i> 32, 837--838.

Sheldrick, G. M. (2008). <i>Acta Cryst.</i> A64, 112--122.

Sheldrick, G. M. (2015). <i>Acta Cryst.</i> C71, 3--8.

Spek, A. L. (2009). <i>Acta Cryst.</i> D65, 148--155.

;

_publ_section_figure_captions

;

;

_publ_section_acknowledgements

;

;

data_new4abovan_0m

_audit_creation_method 'SHELXL-2018/3'

_shelx_SHELXL_version_number '2018/3'

_chemical_name_common ?

_chemical_absolute_configuration '.'

_chemical_name_systematic

;

2-{(E)-[(4-carboxyphenyl)iminio]methyl}-6-methoxyphenolate methanol (1/1)

;

_chemical_formula_moiety 'C15 H13 N O4, C H4 O'

_chemical_formula_sum 'C16 H17 N O5'

_chemical_formula_iupac 'C16 H17 N O5'

_chemical_formula_weight 303.30

loop_

_atom_type_symbol

_atom_type_description

_atom_type_scat_dispersion_real

_atom_type_scat_dispersion_imag

_atom_type_scat_source

'C' 'C' 0.0033 0.0016

'International Tables Vol C Tables 4.2.6.8 and 6.1.1.4'

'H' 'H' 0.0000 0.0000

'International Tables Vol C Tables 4.2.6.8 and 6.1.1.4'

'N' 'N' 0.0061 0.0033

'International Tables Vol C Tables 4.2.6.8 and 6.1.1.4'

'O' 'O' 0.0106 0.0060

'International Tables Vol C Tables 4.2.6.8 and 6.1.1.4'

_space_group_crystal_system orthorhombic

_space_group_IT_number 19

_space_group_name_H-M_alt 'P 21 21 21'

_space_group_name_Hall 'P 2ac 2ab'

_shelx_space_group_comment

;

The symmetry employed for this shelxl refinement is uniquely defined

by the following loop, which should always be used as a source of

symmetry information in preference to the above space-group names.

They are only intended as comments.

;

```
loop_
 _space_group_symop_operation_xyz
 'x, y, z'
 'x+1/2, -y+1/2, -z'
 '-x, y+1/2, -z+1/2'
 '-x+1/2, -y, z+1/2'

_cell_length_a                    4.7768(11)
_cell_length_b                    10.193(3)
_cell_length_c                    30.479(7)
_cell_angle_alpha                 90
_cell_angle_beta                  90
_cell_angle_gamma                 90
_cell_volume                      1484.0(7)
_cell_formula_units_Z             4
_cell_measurement_reflns_used     1418
_cell_measurement_theta_min       2.673
_cell_measurement_theta_max       26.000
```

_cell_measurement_temperature   296(2)

_exptl_crystal_description   needle

_exptl_crystal_colour   'light yellow'

_exptl_crystal_size_max   0.40

_exptl_crystal_size_mid   0.22

_exptl_crystal_size_min   0.16

_exptl_crystal_density_diffrn   1.358

_exptl_crystal_density_meas   ?

_exptl_crystal_density_method   'not measured'

_exptl_crystal_F_000   640

_exptl_absorpt_coefficient_mu   0.102

_exptl_absorpt_correction_type   multi-scan

_exptl_absorpt_process_details   '(SADABS; Bruker, 2005)'

_exptl_absorpt_correction_T_min   0.915

_exptl_absorpt_correction_T_max   0.975

_exptl_special_details

;

;

_diffrn_ambient_temperature   296(2)

_diffrn_radiation_type   MoK\a

_diffrn_radiation_wavelength   0.71073

_diffrn_radiation_source   'fine-focus sealed tube'

_diffrn_radiation_monochromator   graphite

_diffrn_measurement_device_type 'Bruker Kappa APEXII CCD'

_diffrn_measurement_method \w

_diffrn_detector_area_resol_mean 8.225

_diffrn_reflns_number 6629

_diffrn_reflns_av_unetI/netI 0.1486

_diffrn_reflns_av_R_equivalents 0.0816

_diffrn_reflns_limit_h_min -5

_diffrn_reflns_limit_h_max 4

_diffrn_reflns_limit_k_min -10

_diffrn_reflns_limit_k_max 12

_diffrn_reflns_limit_l_min -32

_diffrn_reflns_limit_l_max 36

_diffrn_reflns_theta_min 2.673

_diffrn_reflns_theta_max 25.498

_diffrn_reflns_theta_full 25.242

_diffrn_measured_fraction_theta_max 0.998

_diffrn_measured_fraction_theta_full 0.998

_diffrn_reflns_Laue_measured_fraction_max 0.998

_diffrn_reflns_Laue_measured_fraction_full 0.998

_diffrn_reflns_point_group_measured_fraction_max 0.995

_diffrn_reflns_point_group_measured_fraction_full 0.996

_reflns_Friedel_coverage 0.662

_reflns_Friedel_fraction_max 0.991

_reflns_Friedel_fraction_full 0.993

_diffrn_standards_number 0

_diffrn_standards_interval_count ?

_diffrn_standards_interval_time ?

_diffrn_standards_decay_% ?

_refine_special_details

;

Refinement of <i>F</i>^2^ against ALL reflections. The weighted <i>R</i>-factor

<i>wR</i> and goodness of fit <i>S</i> are based on <i>F</i>^2^, conventional

<i>R</i>-factors <i>R</i> are based on <i>F</i>, with <i>F</i> set to zero for

negative <i>F</i>^2^. The threshold expression of <i>F</i>^2^ >

\s(<i>F</i>^2^) is used only for calculating <i>R</i>-factors(gt) <i>etc</i>.

and is not relevant to the choice of reflections for refinement.

<i>R</i>-factors based on <i>F</i>^2^ are statistically about twice as large

as those based on <i>F</i>, and <i>R</i>- factors based on ALL data will be

even larger.

;

_reflns_number_total 2753

_reflns_number_gt 1398

_reflns_threshold_expression I>2\s(I)

_refine_ls_structure_factor_coef Fsqd

_refine_ls_matrix_type full

_refine_ls_R_factor_all 0.1881

_refine_ls_R_factor_gt 0.0910

_refine_ls_wR_factor_ref 0.1885

_refine_ls_wR_factor_gt 0.1514

_refine_ls_goodness_of_fit_ref 1.087

_refine_ls_restrained_S_all 1.073

_refine_ls_number_reflns 2753

_refine_ls_number_parameters 196

_refine_ls_number_restraints 0

_refine_ls_hydrogen_treatment mixed

_refine_ls_weighting_scheme calc

_refine_ls_weighting_details

'w=1/[\s^2^(Fo^2^)+(0.0439P)^2^+1.0211P] where P=(Fo^2^+2Fc^2^)/3'

_atom_sites_solution_hydrogens geom

_atom_sites_solution_primary direct

_atom_sites_solution_secondary difmap

_refine_ls_extinction_method ?

_refine_ls_extinction_coef ?

_refine_ls_extinction_expression ?

_refine_ls_abs_structure_details

;

Flack x determined using 331 quotients [(I+)-(I-)]/[(I+)+(I-)]

(Parsons, Flack and Wagner, Acta Cryst. B69 (2013) 249-259).

;

_refine_ls_abs_structure_Flack -0.1(10)

_refine_ls_shift/su_max 0.000

_refine_ls_shift/su_mean 0.000

_computing_data_collection 'APEX2 (Bruker, 2007)'

_computing_cell_refinement 'SAINT (Bruker, 2007)'

_computing_data_reduction 'SAINT (Bruker, 2007)'

_computing_structure_solution 'SHELXS97 (Sheldrick, 2008)'

_computing_structure_refinement 'SHELXL-2018/3 (Sheldrick, 2015)'

_computing_molecular_graphics

'ORTEP-3 for Windows (Farrugia, 1997) and PLATON (Spek, 2009)'

_computing_publication_material

;

WinGX (Farrugia, 1999) and PLATON (Spek, 2009)

;

loop_

_atom_site_label

_atom_site_type_symbol

_atom_site_fract_x

_atom_site_fract_y

_atom_site_fract_z

_atom_site_U_iso_or_equiv

_atom_site_adp_type

_atom_site_occupancy

_atom_site_site_symmetry_order

_atom_site_calc_flag

_atom_site_refinement_flags_posn

_atom_site_refinement_flags_adp

_atom_site_refinement_flags_occupancy

_atom_site_disorder_assembly

_atom_site_disorder_group

O1 O -0.0535(16) 0.5295(6) 0.3100(2) 0.070(2) Uani 1 1 d . . . . .

O2 O -0.0383(13) 0.3134(6) 0.3110(2) 0.0505(18) Uani 1 1 d . . . . .

H2 H -0.13(2) 0.323(10) 0.336(3) 0.076 Uiso 1 1 d . U . . .

O3 O 1.0377(11) 0.5583(5) 0.09438(18) 0.0413(16) Uani 1 1 d . U . . .

O4 O 1.4042(11) 0.5791(7) 0.03068(19) 0.0529(18) Uani 1 1 d . U . . .

N1 N 0.7724(14) 0.4142(7) 0.1492(2) 0.0331(17) Uani 1 1 d . . . . .

H1 H 0.823(16) 0.497(8) 0.137(2) 0.040 Uiso 1 1 d . U . . .

C1 C 0.0227(17) 0.4279(9) 0.2938(3) 0.038(2) Uani 1 1 d . . . . .

C2 C 0.1983(9) 0.4190(6) 0.25330(13) 0.032(2) Uani 1 1 d G U . . .

C3 C 0.3135(11) 0.3000(4) 0.24024(16) 0.038(2) Uani 1 1 d G U . . .

H3 H 0.266502 0.223203 0.254998 0.045 Uiso 1 1 calc R U . . .

C4 C 0.4990(11) 0.2957(4) 0.20513(17) 0.041(2) Uani 1 1 d G U . . .

H4 H 0.576100 0.216116 0.196398 0.049 Uiso 1 1 calc R U . . .

C5 C 0.5693(10) 0.4105(5) 0.18308(14) 0.031(2) Uani 1 1 d G U . . .

C6 C 0.4541(11) 0.5294(4) 0.19614(16) 0.042(2) Uani 1 1 d G U . . .

H6 H 0.501094 0.606214 0.181387 0.050 Uiso 1 1 calc R U . . .

C7 C 0.2686(11) 0.5337(4) 0.23125(17) 0.045(3) Uani 1 1 d G U . . .

H7 H 0.191494 0.613303 0.239987 0.054 Uiso 1 1 calc R U . . .

C8 C 0.9209(17) 0.3168(9) 0.1366(3) 0.035(2) Uani 1 1 d . . . . .

H8 H 0.894731 0.235487 0.149823 0.042 Uiso 1 1 calc R U . . .

C9 C 1.1265(16) 0.3296(8) 0.1027(3) 0.033(2) Uani 1 1 d D U . . .

C10 C 1.1721(16) 0.4517(8) 0.0828(3) 0.036(2) Uani 1 1 d D U . . .

C11 C 1.3757(17) 0.4583(9) 0.0487(3) 0.041(2) Uani 1 1 d D U . . .

C12 C 1.5243(17) 0.3477(8) 0.0369(3) 0.044(2) Uani 1 1 d D U . . .

H12 H 1.659643 0.353762 0.015099 0.053 Uiso 1 1 calc R U . . .

C13 C 1.4760(17) 0.2269(9) 0.0571(3) 0.045(2) Uani 1 1 d D U . . .

H13 H 1.576253 0.152965 0.048521 0.054 Uiso 1 1 calc R U . . .

C14 C 1.2779(17) 0.2184(8) 0.0898(3) 0.043(2) Uani 1 1 d D U . . .

H14 H 1.244579 0.138317 0.103512 0.051 Uiso 1 1 calc R U . . .

C15 C 1.614(2) 0.5955(11) -0.0031(3) 0.068(3) Uani 1 1 d . . . . .

H15A H 1.796071 0.577137 0.008912 0.102 Uiso 1 1 calc R U . . .

H15B H 1.609829 0.684097 -0.013762 0.102 Uiso 1 1 calc R U . . .

H15C H 1.576840 0.536140 -0.026823 0.102 Uiso 1 1 calc R U . . .

O5 O 0.2699(14) 0.8018(8) 0.1109(3) 0.067(2) Uani 1 1 d . . . . .

H5A H 0.235257 0.727970 0.101734 0.101 Uiso 1 1 calc R U . . .

C16 C 0.548(2) 0.8372(10) 0.0990(3) 0.067(3) Uani 1 1 d . . . . .

H16A H 0.593617 0.920578 0.111731 0.100 Uiso 1 1 calc R U . . .

H16B H 0.561381 0.842970 0.067613 0.100 Uiso 1 1 calc R U . . .

H16C H 0.675922 0.771833 0.109534 0.100 Uiso 1 1 calc R U . . .

loop_

_atom_site_aniso_label

_atom_site_aniso_U_11

_atom_site_aniso_U_22

_atom_site_aniso_U_33

_atom_site_aniso_U_23

_atom_site_aniso_U_13

_atom_site_aniso_U_12

O1 0.100(6) 0.037(4) 0.074(5) 0.003(4) 0.049(5) 0.008(4)

O2 0.061(5) 0.042(4) 0.049(4) -0.007(3) 0.020(3) -0.005(4)

O3 0.035(4) 0.037(4) 0.051(4) 0.003(3) 0.012(3) 0.005(3)

O4 0.045(4) 0.063(5) 0.050(4) 0.009(4) 0.020(3) 0.003(4)

N1 0.032(4) 0.025(4) 0.042(4) 0.008(4) 0.005(3) -0.009(4)

C1 0.033(5) 0.035(6) 0.047(6) 0.005(5) 0.007(4) 0.005(5)

C2 0.033(5) 0.036(5) 0.027(5) 0.005(4) -0.001(4) -0.010(4)

C3 0.042(6) 0.020(5) 0.051(6) 0.007(4) 0.019(5) -0.008(4)

C4 0.047(6) 0.018(5) 0.057(6) 0.000(4) 0.018(5) 0.008(4)

C5 0.020(5) 0.037(5) 0.035(5) 0.001(4) 0.002(4) -0.007(4)

C6 0.043(6) 0.037(6) 0.044(6) 0.009(4) 0.008(5) -0.007(5)

C7 0.042(6) 0.044(6) 0.048(6) 0.000(5) 0.010(5) 0.007(5)

C8 0.028(5) 0.029(5) 0.047(6) 0.005(4) -0.004(4) -0.008(4)

C9 0.018(4) 0.048(6) 0.033(5) 0.000(4) -0.002(4) -0.008(4)

C10 0.023(5) 0.050(7) 0.035(5) 0.004(5) 0.000(4) -0.007(4)

C11 0.033(5) 0.053(7) 0.037(6) 0.006(5) 0.001(4) -0.004(5)

C12 0.025(5) 0.062(7) 0.046(6) -0.002(5) 0.004(4) -0.009(5)

C13 0.029(5) 0.054(7) 0.051(6) -0.010(5) 0.003(5) 0.003(5)

C14 0.036(6) 0.047(6) 0.045(6) -0.003(5) -0.007(5) -0.008(5)

C15 0.053(7) 0.098(10) 0.053(7) 0.020(7) 0.022(6) 0.005(7)

O5 0.067(5) 0.073(6) 0.062(5) -0.015(4) 0.026(4) -0.015(4)

C16 0.041(7) 0.078(8) 0.081(8) -0.001(7) -0.006(6) -0.005(6)

_geom_special_details

;

All esds (except the esd in the dihedral angle between two l.s. planes)

are estimated using the full covariance matrix. The cell esds are taken
into account individually in the estimation of esds in distances, angles
and torsion angles; correlations between esds in cell parameters are only
used when they are defined by crystal symmetry. An approximate (isotropic)
treatment of cell esds is used for estimating esds involving l.s. planes.
;

```
loop_
 _geom_bond_atom_site_label_1
 _geom_bond_atom_site_label_2
 _geom_bond_distance
 _geom_bond_site_symmetry_2
 _geom_bond_publ_flag
O1 C1 1.203(10) . ?
O2 C1 1.312(10) . ?
O2 H2 0.89(9) . ?
O3 C10 1.310(9) . ?
O4 C11 1.355(9) . ?
O4 C15 1.448(9) . ?
N1 C8 1.279(10) . ?
N1 C5 1.418(7) . ?
N1 H1 0.95(8) . ?
C1 C2 1.495(9) . ?
C2 C3 1.3900 . ?
C2 C7 1.3900 . ?
```

C3 C4 1.3900 . ?

C3 H3 0.9300 . ?

C4 C5 1.3900 . ?

C4 H4 0.9300 . ?

C5 C6 1.3900 . ?

C6 C7 1.3900 . ?

C6 H6 0.9300 . ?

C7 H7 0.9300 . ?

C8 C9 1.432(10) . ?

C8 H8 0.9300 . ?

C9 C10 1.400(8) . ?

C9 C14 1.400(9) . ?

C10 C11 1.426(9) . ?

C11 C12 1.379(9) . ?

C12 C13 1.395(9) . ?

C12 H12 0.9300 . ?

C13 C14 1.378(8) . ?

C13 H13 0.9300 . ?

C14 H14 0.9300 . ?

C15 H15A 0.9600 . ?

C15 H15B 0.9600 . ?

C15 H15C 0.9600 . ?

O5 C16 1.423(10) . ?

O5 H5A 0.8200 . ?

C16 H16A 0.9600 . ?

C16 H16B 0.9600 . ?

C16 H16C 0.9600 . ?

loop_

_geom_angle_atom_site_label_1

_geom_angle_atom_site_label_2

_geom_angle_atom_site_label_3

_geom_angle

_geom_angle_site_symmetry_1

_geom_angle_site_symmetry_3

_geom_angle_publ_flag

C1 O2 H2 111(7) . . ?

C11 O4 C15 117.5(7) . . ?

C8 N1 C5 125.3(7) . . ?

C8 N1 H1 116(5) . . ?

C5 N1 H1 119(5) . . ?

O1 C1 O2 122.3(8) . . ?

O1 C1 C2 124.2(8) . . ?

O2 C1 C2 113.5(8) . . ?

C3 C2 C7 120.0 . . ?

C3 C2 C1 120.8(5) . . ?

C7 C2 C1 118.9(5) . . ?

C2 C3 C4 120.0 . . ?

C2 C3 H3 120.0 . . ?

C4 C3 H3 120.0 . . ?

C5 C4 C3 120.0 . . ?

C5 C4 H4 120.0 . . ?

C3 C4 H4 120.0 . . ?

C4 C5 C6 120.0 . . ?

C4 C5 N1 122.7(5) . . ?

C6 C5 N1 117.1(5) . . ?

C7 C6 C5 120.0 . . ?

C7 C6 H6 120.0 . . ?

C5 C6 H6 120.0 . . ?

C6 C7 C2 120.0 . . ?

C6 C7 H7 120.0 . . ?

C2 C7 H7 120.0 . . ?

N1 C8 C9 121.7(8) . . ?

N1 C8 H8 119.1 . . ?

C9 C8 H8 119.1 . . ?

C10 C9 C14 121.3(7) . . ?

C10 C9 C8 120.0(7) . . ?

C14 C9 C8 118.8(7) . . ?

O3 C10 C9 123.0(7) . . ?

O3 C10 C11 119.4(8) . . ?

C9 C10 C11 117.6(8) . . ?

O4 C11 C12 125.9(7) . . ?

O4 C11 C10 114.0(8) . . ?

C12 C11 C10 120.1(8) . . ?

C11 C12 C13 121.5(8) . . ?

C11 C12 H12 119.3 . . ?

C13 C12 H12 119.3 . . ?

C14 C13 C12 119.2(8) . . ?

C14 C13 H13 120.4 . . ?

C12 C13 H13 120.4 . . ?

C13 C14 C9 120.4(8) . . ?

C13 C14 H14 119.8 . . ?

C9 C14 H14 119.8 . . ?

O4 C15 H15A 109.5 . . ?

O4 C15 H15B 109.5 . . ?

H15A C15 H15B 109.5 . . ?

O4 C15 H15C 109.5 . . ?

H15A C15 H15C 109.5 . . ?

H15B C15 H15C 109.5 . . ?

C16 O5 H5A 109.5 . . ?

O5 C16 H16A 109.5 . . ?

O5 C16 H16B 109.5 . . ?

H16A C16 H16B 109.5 . . ?

O5 C16 H16C 109.5 . . ?

H16A C16 H16C 109.5 . . ?

H16B C16 H16C 109.5 . . ?

loop_

_geom_torsion_atom_site_label_1

_geom_torsion_atom_site_label_2

_geom_torsion_atom_site_label_3

_geom_torsion_atom_site_label_4

_geom_torsion

_geom_torsion_site_symmetry_1

_geom_torsion_site_symmetry_2

_geom_torsion_site_symmetry_3

_geom_torsion_site_symmetry_4

_geom_torsion_publ_flag

O1 C1 C2 C3 170.1(8) . . . . ?

O2 C1 C2 C3 -8.6(9) . . . . ?

O1 C1 C2 C7 -3.4(11) . . . . ?

O2 C1 C2 C7 177.8(5) . . . . ?

C7 C2 C3 C4 0.0 . . . . ?

C1 C2 C3 C4 -173.5(6) . . . . ?

C2 C3 C4 C5 0.0 . . . . ?

C3 C4 C5 C6 0.0 . . . . ?

C3 C4 C5 N1 175.2(5) . . . . ?

C8 N1 C5 C4 -3.5(9) . . . . ?

C8 N1 C5 C6 171.9(6) . . . . ?

C4 C5 C6 C7 0.0 . . . . ?

N1 C5 C6 C7 -175.5(5) . . . . ?

C5 C6 C7 C2 0.0 . . . . ?

C3 C2 C7 C6 0.0 . . . . ?

C1 C2 C7 C6 173.6(6) . . . . ?

C5 N1 C8 C9 -178.6(6) . . . . ?

N1 C8 C9 C10 1.0(11) . . . . ?

N1 C8 C9 C14 -178.7(8) . . . . ?

C14 C9 C10 O3 -178.9(7) . . . . ?

C8 C9 C10 O3 1.4(12) . . . . ?

C14 C9 C10 C11 0.8(12) . . . . ?

C8 C9 C10 C11 -178.9(7) . . . . ?

C15 O4 C11 C12 -1.8(12) . . . . ?

C15 O4 C11 C10 177.7(7) . . . . ?

O3 C10 C11 O4 -1.3(11) . . . . ?

C9 C10 C11 O4 179.0(7) . . . . ?

O3 C10 C11 C12 178.3(7) . . . . ?

C9 C10 C11 C12 -1.5(12) . . . . ?

O4 C11 C12 C13 -179.0(8) . . . . ?

C10 C11 C12 C13 1.5(13) . . . . ?

C11 C12 C13 C14 -0.8(13) . . . . ?

C12 C13 C14 C9 0.1(12) . . . . ?

C10 C9 C14 C13 -0.2(12) . . . . ?

C8 C9 C14 C13 179.5(7) . . . . ?

loop_

_geom_hbond_atom_site_label_D

_geom_hbond_atom_site_label_H

_geom_hbond_atom_site_label_A

_geom_hbond_distance_DH

_geom_hbond_distance_HA

_geom_hbond_distance_DA
_geom_hbond_angle_DHA
_geom_hbond_site_symmetry_A
_geom_hbond_publ_flag
O2 H2 O5 0.89(9) 1.76(9) 2.628(9) 164(10) 3_545 yes
N1 H1 O3 0.95(8) 1.77(8) 2.560(8) 138(7) . yes
C8 H8 O1 0.93 2.55 3.410(11) 154.6 3_645 yes
O5 H5A O3 0.82 1.98 2.765(9) 159.3 1_455 yes

_refine_diff_density_max 0.267
_refine_diff_density_min -0.256
_refine_diff_density_rms 0.059

_shelx_res_file
;
TITL NEW4ABOVAN_0m in P2(1)2(1)2(1) LIGHT YELLOW NEEDLE
shelx.res
created by SHELXL-2018/3 at 12:29:19 on 29-Sep-2021
CELL 0.71073 4.7768 10.1926 30.4787 90.000 90.000 90.000
ZERR 4.00 0.0011 0.0028 0.0074 0.000 0.000 0.000
LATT -1
SYMM x+1/2, -y+1/2, -z
SYMM -x, y+1/2, -z+1/2
SYMM -x+1/2, -y, z+1/2
SFAC C H N O

```
UNIT 64  68  4  20
OMIT -3 51
L.S. 40
ACTA
BOND $H
HTAB
CONF
LIST 4
FMAP 2
PLAN 12
TEMP 23.000
OMIT 0   0   2
OMIT 0   1   1
SIZE 0.40 0.22 0.16
DFIX 1.39 0.01 C9 C10 C10 C11 C11 C12 C12 C13 C13 C14 C14 C9
MPLA 3 C1 O1 O2
MPLA 7 C2 C3 C4 C5 C6 C7 N1
MPLA 10 C8 C9 C10 C11 C12 C13 C14 C15 O3 O4
MPLA 3 C1 O1 O2
SIMU C2 C3 C4 C5 C6 C7 C8
SIMU C9 C10 C11 C12 C13 C14 O3 O4
EQIV $1 -x, y-1/2, -z+1/2
HTAB O2 O5_$1
HTAB N1 O3
EQIV $2 -x+1, y-1/2, -z+1/2
```

```
HTAB C8 O1_$2
EQIV $3 x-1, y, z
HTAB O5 O3_$3
WGHT    0.043900    1.021100
FVAR       0.18742
MOLE 1
O1    4   -0.053492    0.529468    0.310009    11.00000    0.10026    0.03680 =
        0.07438    0.00324    0.04915    0.00824
O2    4   -0.038293    0.313379    0.310956    11.00000    0.06051    0.04236 =
        0.04877   -0.00722    0.01965   -0.00450
H2    2   -0.133385    0.322887    0.335996    11.00000   -1.50000
O3    4    1.037662    0.558311    0.094376    11.00000    0.03509    0.03743 =
        0.05137    0.00305    0.01154    0.00498
O4    4    1.404247    0.579139    0.030683    11.00000    0.04487    0.06329 =
        0.05043    0.00895    0.02032    0.00334
N1    3    0.772379    0.414172    0.149184    11.00000    0.03183    0.02541 =
        0.04220    0.00798    0.00497   -0.00907
H1    2    0.823056    0.496929    0.137213    11.00000   -1.20000
C1    1    0.022713    0.427938    0.293782    11.00000    0.03265    0.03511 =
        0.04696    0.00485    0.00736    0.00514
AFIX  66
C2    1    0.198303    0.418954    0.253303    11.00000    0.03297    0.03629 =
        0.02707    0.00503   -0.00107   -0.01034
C3    1    0.313520    0.299970    0.240244    11.00000    0.04200    0.02044 =
        0.05072    0.00672    0.01911   -0.00842
```

```
AFIX  43
H3    2    0.266502    0.223203    0.254998    11.00000   -1.20000
AFIX  65
C4    1    0.499013    0.295724    0.205135    11.00000    0.04747    0.01794 =
         0.05719   -0.00016    0.01815    0.00813
AFIX  43
H4    2    0.576100    0.216116    0.196398    11.00000   -1.20000
AFIX  65
C5    1    0.569289    0.410462    0.183083    11.00000    0.02000    0.03678 =
         0.03492    0.00054    0.00171   -0.00706
C6    1    0.454074    0.529447    0.196141    11.00000    0.04333    0.03749 =
         0.04378    0.00934    0.00750   -0.00663
AFIX  43
H6    2    0.501094    0.606214    0.181387    11.00000   -1.20000
AFIX  65
C7    1    0.268581    0.533694    0.231251    11.00000    0.04231    0.04412 =
         0.04835   -0.00012    0.01016    0.00712
AFIX  43
H7    2    0.191494    0.613303    0.239987    11.00000   -1.20000
AFIX   0
C8    1    0.920923    0.316787    0.136594    11.00000    0.02801    0.02883 =
         0.04749    0.00471   -0.00379   -0.00826
AFIX  43
H8    2    0.894731    0.235487    0.149823    11.00000   -1.20000
AFIX   0
```

C9 1 1.126534 0.329634 0.102660 11.00000 0.01817 0.04798 =

0.03311 0.00043 -0.00245 -0.00762

C10 1 1.172074 0.451714 0.082824 11.00000 0.02292 0.05023 =

0.03478 0.00414 -0.00028 -0.00722

C11 1 1.375710 0.458284 0.048678 11.00000 0.03339 0.05260 =

0.03747 0.00641 0.00080 -0.00410

C12 1 1.524277 0.347674 0.036935 11.00000 0.02513 0.06232 =

0.04560 -0.00212 0.00374 -0.00874

AFIX 43

H12 2 1.659643 0.353762 0.015099 11.00000 -1.20000

AFIX 0

C13 1 1.475994 0.226866 0.057087 11.00000 0.02889 0.05420 =

0.05112 -0.00980 0.00343 0.00257

AFIX 43

H13 2 1.576253 0.152965 0.048521 11.00000 -1.20000

AFIX 0

C14 1 1.277911 0.218381 0.089842 11.00000 0.03605 0.04723 =

0.04543 -0.00311 -0.00658 -0.00848

AFIX 43

H14 2 1.244579 0.138317 0.103512 11.00000 -1.20000

AFIX 0

C15 1 1.614449 0.595506 -0.003092 11.00000 0.05294 0.09777 =

0.05319 0.01997 0.02208 0.00515

AFIX 137

H15A 2 1.796071 0.577137 0.008912 11.00000 -1.50000

H15B 2 1.609829 0.684097 -0.013762 11.00000 -1.50000

H15C 2 1.576840 0.536140 -0.026823 11.00000 -1.50000

AFIX 0

O5 4 0.269888 0.801822 0.110921 11.00000 0.06654 0.07344 =

0.06184 -0.01518 0.02568 -0.01497

AFIX 147

H5A 2 0.235257 0.727970 0.101734 11.00000 -1.50000

AFIX 0

C16 1 0.547807 0.837177 0.098978 11.00000 0.04146 0.07838 =

0.08055 -0.00145 -0.00639 -0.00504

AFIX 137

H16A 2 0.593617 0.920578 0.111731 11.00000 -1.50000

H16B 2 0.561381 0.842970 0.067613 11.00000 -1.50000

H16C 2 0.675922 0.771833 0.109534 11.00000 -1.50000

AFIX 0

HKLF 4 1 1 0 0 0 1 0 0 0 1

REM NEW4ABOVAN_0m in P2(1)2(1)2(1) LIGHT YELLOW NEEDLE

REM wR2 = 0.1885, GooF = S = 1.087, Restrained GooF = 1.073 for all data

REM R1 = 0.0910 for 1398 Fo > 4sig(Fo) and 0.1881 for all 2753 data

REM 196 parameters refined using 90 restraints

END

WGHT 0.0439 1.0229

REM Instructions for potential hydrogen bonds

HTAB O2 O5_$1

HTAB N1 O3

HTAB C8 O1_$2

HTAB O5 O3_$3

REM Highest difference peak 0.267, deepest hole -0.256, 1-sigma level 0.059

Q1 1 0.4263 0.4836 0.1853 11.00000 0.05 0.27

Q2 1 1.0881 0.6430 0.0906 11.00000 0.05 0.26

Q3 1 1.0095 0.6451 0.0907 11.00000 0.05 0.25

Q4 1 0.2739 0.5382 0.2089 11.00000 0.05 0.24

Q5 1 0.3754 0.3557 0.1985 11.00000 0.05 0.22

Q6 1 1.0265 0.3831 0.0895 11.00000 0.05 0.21

Q7 1 1.2817 0.3873 0.0917 11.00000 0.05 0.21

Q8 1 0.0614 0.5979 0.3050 11.00000 0.05 0.21

Q9 1 0.3387 0.6952 0.0875 11.00000 0.05 0.20

Q10 1 0.5567 0.3944 0.2009 11.00000 0.05 0.20

Q11 1 0.3496 0.3861 0.2546 11.00000 0.05 0.20

Q12 1 0.6706 0.4807 0.1863 11.00000 0.05 0.19

;

CIF of DHBA without hkl data

data_global

#==

PROCESSING SUMMARY (IUCr Office Use only)

_publ_contact_author

;

Muhammad Nawaz Tahir

University of Sargodha

Department of Physics

Sargodha

Pakistan

;

_publ_contact_author_phone '0092 48 92 30 914'

_publ_contact_author_fax '0092 48 32 22 121'

_publ_contact_author_email 'dmntahir_uos@yahoo.com'

_publ_requested_journal ''

_journal_date_recd_electronic ?

_journal_date_to_coeditor ?

_journal_date_from_coeditor ?

_journal_date_accepted ?

_journal_date_printers_first ?

_journal_date_printers_final ?

```
_journal_date_proofs_out          ?
_journal_date_proofs_in           ?

_journal_coeditor_name            ?
_journal_coeditor_code     ?
_journal_paper_category      ?
_journal_coeditor_notes
;
;

_journal_techeditor_code      ?
_iucr_compatibility_tag          ?
_journal_techeditor_notes
;
;

_journal_coden_ASTM               ?
_journal_name_full                ?
_journal_year                     ?
_journal_volume                   ?
_journal_issue                    ?
_journal_page_first               ?
_journal_page_last                ?

_journal_suppl_publ_number        ?
```

_journal_suppl_publ_pages ?
#==

loop_
_publ_author_name
_publ_author_address

'Muhammad Nawaz Tahir'
;
Department of Physics
University of Sargodha
Sargodha
Pakistan
;

'Muhammad Ashfaq'
;
Department of Physics
University of Sargodha
Sargodha
Pakistan
;

'Akbar Ali'
;

Department of Chemistry,

Government College University,

Faisalabad,

Pakistan

;

'Muhammad Khalid'

;

Department of Chemistry,

Khwaja Fareed University of Engineering and Information Technology,

Rahim Yar Khan,64200,

Pakistan

;

_audit_creation_date ?

_audit_creation_method ?

#===

TEXT

_publ_section_title

;

(E)-4-((2-hydroxy-3,5-diiodobenzylidene)amino)benzoic acid (methylsulfinyl)

methane solvate (1:1)

;

_publ_section_abstract

;

;

_publ_section_comment

;

;

_publ_section_exptl_refinement

;

;

_publ_section_exptl_prep

;

;

_publ_section_related_literature

;

;

_publ_section_references

;

Bruker (2005). <i>SADABS</i>. Bruker AXS Inc., Madison, Wisconsin, USA.

Bruker (2007). <i>APEX2</i> and <i>SAINT</i>. Bruker AXS Inc.,
Madison, Wisconsin, USA.

Farrugia, L. J. (1997). <i>J. Appl. Cryst.</i> 30, 565.

Farrugia, L. J. (1999). <i>J. Appl. Cryst.</i> 32, 837--838.

Sheldrick, G. M. (2008). <i>Acta Cryst.</i> A64, 112--122.

Sheldrick, G. M. (2015). <i>Acta Cryst.</i> C71, 3--8.

Spek, A. L. (2009). <i>Acta Cryst.</i> D65, 148--155.
;

_publ_section_figure_captions
;
;
_publ_section_acknowledgements
;
;

data_4aba35diiodo

_audit_creation_method 'SHELXL-2018/3'

_shelx_SHELXL_version_number '2018/3'

_chemical_name_common ?

_chemical_absolute_configuration '.'

_chemical_name_systematic

;

(E)-4-((2-hydroxy-3,5-diiodobenzylidene)amino)benzoic acid (methylsulfinyl)

methane solvate (1:1)

;

_chemical_formula_moiety 'C16 H15 I2 N O4 S'

_chemical_formula_sum 'C16 H15 I2 N O4 S'

_chemical_formula_iupac 'C16 H15 I2 N O4 S'

_chemical_formula_weight 571.15

loop_

_atom_type_symbol

_atom_type_description

_atom_type_scat_dispersion_real

_atom_type_scat_dispersion_imag

_atom_type_scat_source

'C' 'C' 0.0033 0.0016

'International Tables Vol C Tables 4.2.6.8 and 6.1.1.4'

'H' 'H' 0.0000 0.0000

'International Tables Vol C Tables 4.2.6.8 and 6.1.1.4'

'N' 'N'   0.0061   0.0033

'International Tables Vol C Tables 4.2.6.8 and 6.1.1.4'

'O' 'O'   0.0106   0.0060

'International Tables Vol C Tables 4.2.6.8 and 6.1.1.4'

'S' 'S'   0.1246   0.1234

'International Tables Vol C Tables 4.2.6.8 and 6.1.1.4'

'I' 'I' -0.4742   1.8119

'International Tables Vol C Tables 4.2.6.8 and 6.1.1.4'

_space_group_crystal_system   monoclinic

_space_group_IT_number   14

_space_group_name_H-M_alt   'P 21/c'

_space_group_name_Hall   '-P 2ybc'

_shelx_space_group_comment

;

The symmetry employed for this shelxl refinement is uniquely defined

by the following loop, which should always be used as a source of

symmetry information in preference to the above space-group names.

They are only intended as comments.

;

loop_

_space_group_symop_operation_xyz

'x, y, z'

'-x, y+1/2, -z+1/2'

'-x, -y, -z'

'x, -y-1/2, z-1/2'

_cell_length_a 21.441(4)

_cell_length_b 5.2059(9)

_cell_length_c 17.465(4)

_cell_angle_alpha 90

_cell_angle_beta 102.673(10)

_cell_angle_gamma 90

_cell_volume 1901.9(7)

_cell_formula_units_Z 4

_cell_measurement_reflns_used 2516

_cell_measurement_theta_min 2.921

_cell_measurement_theta_max 27.000

_cell_measurement_temperature 296(2)

_exptl_crystal_description needle

_exptl_crystal_colour 'orange'

_exptl_crystal_size_max 0.43

_exptl_crystal_size_mid 0.20

_exptl_crystal_size_min 0.14

_exptl_crystal_density_diffrn 1.995

_exptl_crystal_density_meas ?

_exptl_crystal_density_method 'not measured'

_exptl_crystal_F_000 1088

_exptl_absorpt_coefficient_mu 3.435

_exptl_absorpt_correction_type multi-scan

_exptl_absorpt_process_details '(SADABS; Bruker, 2005)'

_exptl_absorpt_correction_T_min 0.945

_exptl_absorpt_correction_T_max 0.985

_exptl_special_details

;

;

_diffrn_ambient_temperature 296(2)

_diffrn_radiation_type MoK\a

_diffrn_radiation_wavelength 0.71073

_diffrn_radiation_source 'fine-focus sealed tube'

_diffrn_radiation_monochromator graphite

_diffrn_measurement_device_type 'Bruker Kappa APEXII CCD'

_diffrn_measurement_method \w

_diffrn_detector_area_resol_mean 7.83

_diffrn_reflns_number 11274

_diffrn_reflns_av_unetI/netI 0.0652

_diffrn_reflns_av_R_equivalents 0.0492

_diffrn_reflns_limit_h_min -25

_diffrn_reflns_limit_h_max 27

_diffrn_reflns_limit_k_min -6

_diffrn_reflns_limit_k_max 6

_diffrn_reflns_limit_l_min -16

_diffrn_reflns_limit_l_max 22

_diffrn_reflns_theta_min 2.921

_diffrn_reflns_theta_max 27.000

_diffrn_reflns_theta_full 25.242

_diffrn_measured_fraction_theta_max 0.994

_diffrn_measured_fraction_theta_full 0.994

_diffrn_standards_number 0

_diffrn_standards_interval_count ?

_diffrn_standards_interval_time ?

_diffrn_standards_decay_% ?

_refine_special_details

;

Refinement of <i>F</i>^2^ against ALL reflections. The weighted <i>R</i>-factor

<i>wR</i> and goodness of fit <i>S</i> are based on <i>F</i>^2^, conventional

<i>R</i>-factors <i>R</i> are based on <i>F</i>, with <i>F</i> set to zero for

negative <i>F</i>^2^. The threshold expression of <i>F</i>^2^ >

\s(<i>F</i>^2^) is used only for calculating <i>R</i>-factors(gt) <i>etc</i>.

and is not relevant to the choice of reflections for refinement.

<i>R</i>-factors based on <i>F</i>^2^ are statistically about twice as large

as those based on <i>F</i>, and <i>R</i>- factors based on ALL data will be

even larger.

;

_reflns_number_total 4128

_reflns_number_gt 2516

_reflns_threshold_expression I>2\s(I)

_refine_ls_structure_factor_coef Fsqd

_refine_ls_matrix_type full

_refine_ls_R_factor_all 0.0865

_refine_ls_R_factor_gt 0.0439

_refine_ls_wR_factor_ref 0.1028

_refine_ls_wR_factor_gt 0.0854

_refine_ls_goodness_of_fit_ref 1.008

_refine_ls_restrained_S_all 1.008

_refine_ls_number_reflns 4128

_refine_ls_number_parameters 186

_refine_ls_number_restraints 0

_refine_ls_hydrogen_treatment constr

_refine_ls_weighting_scheme calc

_refine_ls_weighting_details

'w=1/[\s^2^(Fo^2^)+(0.0335P)^2^+0.5542P] where P=(Fo^2^+2Fc^2^)/3'

_atom_sites_solution_hydrogens geom

_atom_sites_solution_primary direct

_atom_sites_solution_secondary difmap

_refine_ls_extinction_method ?

_refine_ls_extinction_coef ?

_refine_ls_extinction_expression ?

_refine_ls_shift/su_max 0.001

_refine_ls_shift/su_mean 0.000

_computing_data_collection 'APEX2 (Bruker, 2007)'

_computing_cell_refinement 'SAINT (Bruker, 2007)'

_computing_data_reduction 'SAINT (Bruker, 2007)'

_computing_structure_solution 'SHELXS97 (Sheldrick, 2008)'

_computing_structure_refinement 'SHELXL-2018/3 (Sheldrick, 2015)'

_computing_molecular_graphics

'ORTEP-3 for Windows (Farrugia, 1997) and PLATON (Spek, 2009)'

_computing_publication_material

;

WinGX (Farrugia, 1999) and PLATON (Spek, 2009)

;

loop_

_atom_site_label

_atom_site_type_symbol

_atom_site_fract_x

_atom_site_fract_y

_atom_site_fract_z

_atom_site_U_iso_or_equiv

_atom_site_adp_type

_atom_site_occupancy

_atom_site_site_symmetry_order

_atom_site_calc_flag

_atom_site_refinement_flags_posn

_atom_site_refinement_flags_adp

_atom_site_refinement_flags_occupancy

_atom_site_disorder_assembly

_atom_site_disorder_group

I1 I 0.45875(2) 0.39458(8) 0.66274(3) 0.07566(18) Uani 1 1 d . . . . .

I2 I 0.39585(2) 1.15348(7) 0.39836(3) 0.06292(16) Uani 1 1 d . . . . .

O1 O -0.1232(2) 0.4065(8) 0.3062(3) 0.0895(16) Uani 1 1 d . . . . .

O2 O -0.0980(2) 0.1275(10) 0.4028(3) 0.1002(18) Uani 1 1 d . . . . .

H2 H -0.136604 0.100902 0.391981 0.150 Uiso 1 1 calc R U . . .

O3 O 0.25903(17) 0.9415(7) 0.4034(2) 0.0587(10) Uani 1 1 d . . . . .

H3 H 0.223899 0.873688 0.398937 0.088 Uiso 1 1 calc R U . . .

N1 N 0.1740(2) 0.6403(8) 0.4369(3) 0.0510(11) Uani 1 1 d . . . . .

C1 C -0.0845(3) 0.3150(12) 0.3590(4) 0.0647(17) Uani 1 1 d . . . . .

C2A C -0.0156(3) 0.3841(16) 0.3762(5) 0.0532(8) Uani 0.538(13) 1 d G . P A 1

C3A C 0.0298(4) 0.2338(16) 0.4255(6) 0.0532(8) Uani 0.538(13) 1 d G . P A 1

H3A H 0.017819 0.079068 0.444734 0.064 Uiso 0.538(13) 1 calc R U P A 1

C4A C 0.0930(3) 0.3147(17) 0.4460(6) 0.0532(8) Uani 0.538(13) 1 d G . P A 1

H4A H 0.123401 0.214096 0.478992 0.064 Uiso 0.538(13) 1 calc R U P A 1

C5A C 0.1109(3) 0.5459(15) 0.4173(5) 0.0532(8) Uani 0.538(13) 1 d G . P A 1

C6A C 0.0655(4) 0.6962(13) 0.3680(6) 0.0532(8) Uani 0.538(13) 1 d G . P A 1

H6A H 0.077490 0.850917 0.348811 0.064 Uiso 0.538(13) 1 calc R U P A 1

C7A C 0.0023(4) 0.6153(15) 0.3475(5) 0.0532(8) Uani 0.538(13) 1 d G . P A 1

H7A H -0.028092 0.715893 0.314552 0.064 Uiso 0.538(13) 1 calc R U P A 1

C2B C -0.0176(3) 0.4016(18) 0.3829(6) 0.0532(8) Uani 0.462(13) 1 d G . P A 2

C3B C 0.0222(4) 0.316(2) 0.4520(6) 0.0532(8) Uani 0.462(13) 1 d G . P A 2

H3B H 0.006099 0.208312 0.485645 0.064 Uiso 0.462(13) 1 calc R U P A 2

C4B C 0.0860(4) 0.390(2) 0.4709(6) 0.0532(8) Uani 0.462(13) 1 d G . P A 2

H4B H 0.112558 0.332907 0.517125 0.064 Uiso 0.462(13) 1 calc R U P A 2

C5B C 0.1100(3) 0.5509(17) 0.4206(6) 0.0532(8) Uani 0.462(13) 1 d G . P A 2

C6B C 0.0702(5) 0.637(2) 0.3515(6) 0.0532(8) Uani 0.462(13) 1 d G . P A 2

H6B H 0.086272 0.744289 0.317931 0.064 Uiso 0.462(13) 1 calc R U P A 2

C7B C 0.0064(5) 0.562(2) 0.3327(5) 0.0532(8) Uani 0.462(13) 1 d G . P A 2

H7B H -0.020186 0.619695 0.286449 0.064 Uiso 0.462(13) 1 calc R U P A 2

C8 C 0.2174(3) 0.5400(11) 0.4899(4) 0.0546(14) Uani 1 1 d . . . . .

H8 H 0.206455 0.408753 0.520575 0.065 Uiso 1 1 calc R U . . .

C9 C 0.2824(2) 0.6230(9) 0.5036(3) 0.0475(13) Uani 1 1 d . . . . .

C10 C 0.3011(2) 0.8222(9) 0.4582(3) 0.0464(13) Uani 1 1 d . . . . .

C11 C 0.3660(2) 0.8848(8) 0.4711(3) 0.0441(13) Uani 1 1 d . . . . .

C12 C 0.4104(2) 0.7678(9) 0.5286(3) 0.0495(13) Uani 1 1 d . . . . .

H12 H 0.453215 0.814285 0.536611 0.059 Uiso 1 1 calc R U . . .

C13 C 0.3915(2) 0.5791(9) 0.5749(3) 0.0510(14) Uani 1 1 d . . . . .

C14 C 0.3285(3) 0.5055(11) 0.5608(3) 0.0532(14) Uani 1 1 d . . . . .

H14 H 0.316472 0.373429 0.590405 0.064 Uiso 1 1 calc R U . . .

S1 S 0.25533(7) 0.3760(3) 0.20204(10) 0.0595(4) Uani 1 1 d . . . . .

O4 O 0.21352(19) 0.4436(8) 0.1234(2) 0.0776(13) Uani 1 1 d . . . . .

C15 C 0.2085(3) 0.3928(11) 0.2726(4) 0.0736(18) Uani 1 1 d . . . . .

H15A H 0.234683 0.357284 0.323515 0.110 Uiso 1 1 calc R U . . .

H15B H 0.174689 0.268631 0.260502 0.110 Uiso 1 1 calc R U . . .

H15C H 0.190561 0.561848 0.272309 0.110 Uiso 1 1 calc R U . . .

C16 C 0.3032(4) 0.6479(12) 0.2314(5) 0.104(3) Uani 1 1 d . . . . .

H16A H 0.337971 0.649890 0.204937 0.157 Uiso 1 1 calc R U . . .

H16B H 0.319851 0.641889 0.287121 0.157 Uiso 1 1 calc R U . . .

H16C H 0.277934 0.800356 0.218342 0.157 Uiso 1 1 calc R U . . .

loop_

_atom_site_aniso_label

_atom_site_aniso_U_11

_atom_site_aniso_U_22

_atom_site_aniso_U_33

_atom_site_aniso_U_23

_atom_site_aniso_U_13

_atom_site_aniso_U_12

I1 0.0621(3) 0.0898(3) 0.0635(3) 0.0171(2) -0.0114(2) 0.0019(2)

I2 0.0504(2) 0.0675(3) 0.0693(3) 0.0146(2) 0.0097(2) -0.00866(17)

O1 0.053(3) 0.090(3) 0.111(4) 0.019(3) -0.012(3) -0.012(2)

O2 0.059(3) 0.141(4) 0.090(4) 0.033(3) -0.007(3) -0.042(3)

O3 0.046(2) 0.066(2) 0.060(3) 0.014(2) 0.002(2) -0.0052(18)

N1 0.040(3) 0.069(3) 0.045(3) -0.003(2) 0.011(2) -0.005(2)

C1 0.053(4) 0.080(4) 0.059(5) -0.002(4) 0.008(3) -0.014(3)

C2A 0.0469(15) 0.0647(19) 0.047(2) -0.0015(14) 0.0073(13) -0.0040(13)

C3A 0.0469(15) 0.0647(19) 0.047(2) -0.0015(14) 0.0073(13) -0.0040(13)

C4A 0.0469(15) 0.0647(19) 0.047(2) -0.0015(14) 0.0073(13) -0.0040(13)

C5A 0.0469(15) 0.0647(19) 0.047(2) -0.0015(14) 0.0073(13) -0.0040(13)

C6A 0.0469(15) 0.0647(19) 0.047(2) -0.0015(14) 0.0073(13) -0.0040(13)

C7A 0.0469(15) 0.0647(19) 0.047(2) -0.0015(14) 0.0073(13) -0.0040(13)

C2B 0.0469(15) 0.0647(19) 0.047(2) -0.0015(14) 0.0073(13) -0.0040(13)

C3B 0.0469(15) 0.0647(19) 0.047(2) -0.0015(14) 0.0073(13) -0.0040(13)

C4B 0.0469(15) 0.0647(19) 0.047(2) -0.0015(14) 0.0073(13) -0.0040(13)

C5B 0.0469(15) 0.0647(19) 0.047(2) -0.0015(14) 0.0073(13) -0.0040(13)

C6B 0.0469(15) 0.0647(19) 0.047(2) -0.0015(14) 0.0073(13) -0.0040(13)

C7B 0.0469(15) 0.0647(19) 0.047(2) -0.0015(14) 0.0073(13) -0.0040(13)

C8 0.045(3) 0.068(3) 0.051(4) 0.004(3) 0.011(3) -0.007(3)

C9 0.037(3) 0.066(3) 0.041(3) -0.002(3) 0.009(3) 0.000(2)

C10 0.044(3) 0.051(3) 0.041(3) 0.001(3) 0.003(3) 0.003(2)

C11 0.038(3) 0.049(3) 0.045(4) -0.003(2) 0.007(3) -0.002(2)

C12 0.037(3) 0.058(3) 0.052(4) -0.002(3) 0.007(3) -0.004(2)

C13 0.045(3) 0.059(3) 0.046(4) 0.002(3) 0.002(3) 0.001(2)

C14 0.057(4) 0.068(3) 0.036(4) 0.007(3) 0.014(3) -0.003(3)

S1 0.0603(9) 0.0607(9) 0.0553(10) -0.0049(7) 0.0079(8) 0.0158(7)

O4 0.063(3) 0.117(3) 0.050(3) -0.007(2) 0.005(2) 0.031(2)

C15 0.077(5) 0.088(5) 0.056(4) 0.003(3) 0.014(4) 0.001(3)

C16 0.120(7) 0.096(5) 0.093(7) 0.012(4) 0.013(5) -0.040(4)

_geom_special_details

;

All esds (except the esd in the dihedral angle between two l.s. planes)

are estimated using the full covariance matrix. The cell esds are taken

into account individually in the estimation of esds in distances, angles

and torsion angles; correlations between esds in cell parameters are only

used when they are defined by crystal symmetry. An approximate (isotropic)

treatment of cell esds is used for estimating esds involving l.s. planes.

;

loop_
 _geom_bond_atom_site_label_1
 _geom_bond_atom_site_label_2
 _geom_bond_distance
 _geom_bond_site_symmetry_2
 _geom_bond_publ_flag
I1 C13 2.095(5) . ?
I2 C11 2.083(5) . ?
O1 C1 1.196(7) . ?
O2 C1 1.311(7) . ?
O2 H2 0.8200 . ?
O3 C10 1.318(6) . ?
O3 H3 0.8200 . ?
N1 C8 1.271(7) . ?
N1 C5A 1.410(7) . ?
N1 C5B 1.419(8) . ?
C1 C2B 1.473(9) . ?
C1 C2A 1.485(8) . ?
C2A C3A 1.3900 . ?
C2A C7A 1.3900 . ?
C3A C4A 1.3900 . ?
C3A H3A 0.9300 . ?
C4A C5A 1.3900 . ?

C4A H4A 0.9300 . ?

C5A C6A 1.3900 . ?

C6A C7A 1.3900 . ?

C6A H6A 0.9300 . ?

C7A H7A 0.9300 . ?

C2B C3B 1.3900 . ?

C2B C7B 1.3900 . ?

C3B C4B 1.3900 . ?

C3B H3B 0.9300 . ?

C4B C5B 1.3900 . ?

C4B H4B 0.9300 . ?

C5B C6B 1.3900 . ?

C6B C7B 1.3900 . ?

C6B H6B 0.9300 . ?

C7B H7B 0.9300 . ?

C8 C9 1.429(7) . ?

C8 H8 0.9300 . ?

C9 C14 1.383(7) . ?

C9 C10 1.416(7) . ?

C10 C11 1.398(7) . ?

C11 C12 1.366(7) . ?

C12 C13 1.388(7) . ?

C12 H12 0.9300 . ?

C13 C14 1.373(7) . ?

C14 H14 0.9300 . ?

S1 O4 1.508(4) . ?

S1 C15 1.754(6) . ?

S1 C16 1.758(6) . ?

C15 H15A 0.9600 . ?

C15 H15B 0.9600 . ?

C15 H15C 0.9600 . ?

C16 H16A 0.9600 . ?

C16 H16B 0.9600 . ?

C16 H16C 0.9600 . ?

loop_

_geom_angle_atom_site_label_1

_geom_angle_atom_site_label_2

_geom_angle_atom_site_label_3

_geom_angle

_geom_angle_site_symmetry_1

_geom_angle_site_symmetry_3

_geom_angle_publ_flag

C1 O2 H2 109.5 . . ?

C10 O3 H3 109.5 . . ?

C8 N1 C5A 122.9(5) . . ?

C8 N1 C5B 122.1(6) . . ?

O1 C1 O2 122.7(6) . . ?

O1 C1 C2B 124.4(7) . . ?

O2 C1 C2B 112.8(7) . . ?

O1 C1 C2A 123.5(7) . . ?

O2 C1 C2A 113.5(6) . . ?

C3A C2A C7A 120.0 . . ?

C3A C2A C1 121.3(6) . . ?

C7A C2A C1 118.5(6) . . ?

C2A C3A C4A 120.0 . . ?

C2A C3A H3A 120.0 . . ?

C4A C3A H3A 120.0 . . ?

C5A C4A C3A 120.0 . . ?

C5A C4A H4A 120.0 . . ?

C3A C4A H4A 120.0 . . ?

C6A C5A C4A 120.0 . . ?

C6A C5A N1 117.3(6) . . ?

C4A C5A N1 122.7(6) . . ?

C5A C6A C7A 120.0 . . ?

C5A C6A H6A 120.0 . . ?

C7A C6A H6A 120.0 . . ?

C6A C7A C2A 120.0 . . ?

C6A C7A H7A 120.0 . . ?

C2A C7A H7A 120.0 . . ?

C3B C2B C7B 120.0 . . ?

C3B C2B C1 121.4(7) . . ?

C7B C2B C1 118.5(7) . . ?

C4B C3B C2B 120.0 . . ?

C4B C3B H3B 120.0 . . ?

C2B C3B H3B 120.0 . . ?

C3B C4B C5B 120.0 . . ?

C3B C4B H4B 120.0 . . ?

C5B C4B H4B 120.0 . . ?

C6B C5B C4B 120.0 . . ?

C6B C5B N1 116.8(7) . . ?

C4B C5B N1 123.2(7) . . ?

C5B C6B C7B 120.0 . . ?

C5B C6B H6B 120.0 . . ?

C7B C6B H6B 120.0 . . ?

C6B C7B C2B 120.0 . . ?

C6B C7B H7B 120.0 . . ?

C2B C7B H7B 120.0 . . ?

N1 C8 C9 121.9(5) . . ?

N1 C8 H8 119.0 . . ?

C9 C8 H8 119.0 . . ?

C14 C9 C10 118.9(5) . . ?

C14 C9 C8 120.2(5) . . ?

C10 C9 C8 120.8(5) . . ?

O3 C10 C11 120.4(5) . . ?

O3 C10 C9 121.3(5) . . ?

C11 C10 C9 118.3(5) . . ?

C12 C11 C10 121.6(5) . . ?

C12 C11 I2 119.3(4) . . ?

C10 C11 I2 119.1(4) . . ?

C11 C12 C13 119.8(5) . . ?

C11 C12 H12 120.1 . . ?

C13 C12 H12 120.1 . . ?

C14 C13 C12 119.7(5) . . ?

C14 C13 I1 119.7(4) . . ?

C12 C13 I1 120.6(4) . . ?

C13 C14 C9 121.6(5) . . ?

C13 C14 H14 119.2 . . ?

C9 C14 H14 119.2 . . ?

O4 S1 C15 108.1(3) . . ?

O4 S1 C16 105.7(3) . . ?

C15 S1 C16 98.2(4) . . ?

S1 C15 H15A 109.5 . . ?

S1 C15 H15B 109.5 . . ?

H15A C15 H15B 109.5 . . ?

S1 C15 H15C 109.5 . . ?

H15A C15 H15C 109.5 . . ?

H15B C15 H15C 109.5 . . ?

S1 C16 H16A 109.5 . . ?

S1 C16 H16B 109.5 . . ?

H16A C16 H16B 109.5 . . ?

S1 C16 H16C 109.5 . . ?

H16A C16 H16C 109.5 . . ?

H16B C16 H16C 109.5 . . ?

loop_

_geom_torsion_atom_site_label_1

_geom_torsion_atom_site_label_2

_geom_torsion_atom_site_label_3

_geom_torsion_atom_site_label_4

_geom_torsion

_geom_torsion_site_symmetry_1

_geom_torsion_site_symmetry_2

_geom_torsion_site_symmetry_3

_geom_torsion_site_symmetry_4

_geom_torsion_publ_flag

O1 C1 C2A C3A -164.9(7) . . . . ?

O2 C1 C2A C3A 9.4(9) . . . . ?

O1 C1 C2A C7A 20.5(10) . . . . ?

O2 C1 C2A C7A -165.1(7) . . . . ?

C7A C2A C3A C4A 0.0 . . . . ?

C1 C2A C3A C4A -174.4(7) . . . . ?

C2A C3A C4A C5A 0.0 . . . . ?

C3A C4A C5A C6A 0.0 . . . . ?

C3A C4A C5A N1 179.0(7) . . . . ?

C8 N1 C5A C6A 169.5(6) . . . . ?

C8 N1 C5A C4A -9.6(9) . . . . ?

C4A C5A C6A C7A 0.0 . . . . ?

N1 C5A C6A C7A -179.1(7) . . . . ?

C5A C6A C7A C2A 0.0 . . . . ?

C3A C2A C7A C6A 0.0 . . . . ?

C1 C2A C7A C6A 174.6(7) . . . . ?

O1 C1 C2B C3B 169.7(7) . . . . ?

O2 C1 C2B C3B -9.1(9) . . . . ?

O1 C1 C2B C7B -13.7(11) . . . . ?

O2 C1 C2B C7B 167.5(7) . . . . ?

C7B C2B C3B C4B 0.0 . . . . ?

C1 C2B C3B C4B 176.6(8) . . . . ?

C2B C3B C4B C5B 0.0 . . . . ?

C3B C4B C5B C6B 0.0 . . . . ?

C3B C4B C5B N1 178.4(8) . . . . ?

C8 N1 C5B C6B -166.8(6) . . . . ?

C8 N1 C5B C4B 14.7(10) . . . . ?

C4B C5B C6B C7B 0.0 . . . . ?

N1 C5B C6B C7B -178.5(8) . . . . ?

C5B C6B C7B C2B 0.0 . . . . ?

C3B C2B C7B C6B 0.0 . . . . ?

C1 C2B C7B C6B -176.7(8) . . . . ?

C5A N1 C8 C9 173.9(6) . . . . ?

C5B N1 C8 C9 177.2(6) . . . . ?

N1 C8 C9 C14 -178.7(5) . . . . ?

N1 C8 C9 C10 0.0(8) . . . . ?

C14 C9 C10 O3 -179.5(5) . . . . ?

C8 C9 C10 O3 1.8(8) . . . . ?

C14 C9 C10 C11 2.4(8) . . . . ?

C8 C9 C10 C11 -176.2(5) . . . . ?

O3 C10 C11 C12 178.7(5) . . . . ?

C9 C10 C11 C12 -3.2(8) . . . . ?

O3 C10 C11 I2 -3.6(7) . . . . ?

C9 C10 C11 I2 174.5(4) . . . . ?

C10 C11 C12 C13 0.9(8) . . . . ?

I2 C11 C12 C13 -176.8(4) . . . . ?

C11 C12 C13 C14 2.2(8) . . . . ?

C11 C12 C13 I1 179.5(4) . . . . ?

C12 C13 C14 C9 -3.0(8) . . . . ?

I1 C13 C14 C9 179.7(4) . . . . ?

C10 C9 C14 C13 0.7(8) . . . . ?

C8 C9 C14 C13 179.3(5) . . . . ?

loop_

_geom_hbond_atom_site_label_D

_geom_hbond_atom_site_label_H

_geom_hbond_atom_site_label_A

_geom_hbond_distance_DH

_geom_hbond_distance_HA

_geom_hbond_distance_DA

_geom_hbond_angle_DHA

_geom_hbond_site_symmetry_A

_geom_hbond_publ_flag

O2 H2 S1 0.82 2.95 3.707(4) 154.1 2_545 yes

O2 H2 O4 0.82 1.81 2.601(5) 162.4 2_545 yes

O3 H3 N1 0.82 1.84 2.567(5) 147.9 . yes

C6B^b H6B^b O1 0.93 2.61 3.496(9) 160.4 2 yes

C8 H8 O4 0.93 2.55 3.445(7) 162.2 4_566 yes

C12 H12 I2 0.93 3.19 4.090(5) 163.4 3_676 yes

C14 H14 S1 0.93 2.89 3.767(6) 158.5 4_566 yes

C15 H15A O3 0.96 2.57 3.290(7) 132.4 1_545 yes

C15 H15B O1 0.96 2.36 3.247(7) 153.6 2_545 yes

C15 H15C O1 0.96 2.51 3.359(7) 147.0 2 yes

_refine_diff_density_max   0.868

_refine_diff_density_min   -0.754

_refine_diff_density_rms   0.105

_shelx_res_file

;

TITL 4ABA35DIIODO in P2(1)/c ORANGE NEEDLE

shelx.res

created by SHELXL-2018/3 at 14:44:43 on 31-Jan-2021

CELL 0.71073 21.4407   5.2059 17.4652 90.000 102.673 90.000

ZERR   4.00   0.0039   0.0009   0.0040   0.000   0.010   0.000

LATT 1

SYMM -x, y+1/2, -z+1/2

SFAC C H N O S I

```
UNIT 64  60  4  16  4  8
OMIT -3 54
L.S. 40
ACTA
BOND $H
HTAB
CONF
LIST 4
FMAP 2
PLAN 15
TEMP 23.000
SIZE 0.43 0.20 0.14
OMIT-2   0   2
OMIT 2   0   0
OMIT 1   0   0
OMIT-1   0   2
EADP C2A C3A C4A C5A C6A C7A C2B C3B C4B C5B C6B C7B
EQIV $1 -x, y-1/2, -z+1/2
HTAB O2 S1_$1
HTAB O2 O4_$1
HTAB O3 N1
EQIV $2 -x, y+1/2, -z+1/2
HTAB C6B O1_$2
EQIV $3 x, -y+1/2, z+1/2
HTAB C8 O4_$3
```

```
EQIV $4 -x+1, -y+2, -z+1
HTAB C12 I2_$4
HTAB C14 S1_$3
EQIV $5 x, y-1, z
HTAB C15 O3_$5
HTAB C15 O1_$1
HTAB C15 O1_$2
MPLA 3 C1 O1 O2
MPLA 7 C2A C3A C4A C5A C6A C7A N1
MPLA 7 C2B C3B C4B C5B C6B C7B N1
MPLA 3 C1 O1 O2
MPLA 10 C8 C9 C10 C11 C12 C13 C14 O3 I1 I2
MPLA 7 C2A C3A C4A C5A C6A C7A N1
MPLA 7 C2B C3B C4B C5B C6B C7B N1
MPLA 10 C8 C9 C10 C11 C12 C13 C14 O3 I1 I2
WGHT    0.033500    0.554200
FVAR       0.07675   0.53764
MOLE 1
I1    6    0.458755    0.394577    0.662741    11.00000    0.06211    0.08982 =
       0.06346    0.01710   -0.01138    0.00191
I2    6    0.395846    1.153480    0.398364    11.00000    0.05036    0.06752 =
       0.06930    0.01461    0.00970   -0.00866
O1    4   -0.123202    0.406475    0.306199    11.00000    0.05340    0.08973 =
       0.11142    0.01883   -0.01227   -0.01224
O2    4   -0.097969    0.127539    0.402760    11.00000    0.05928    0.14127 =
```

0.08960 0.03291 -0.00664 -0.04184

AFIX 147

H2 2 -0.136604 0.100902 0.391981 11.00000 -1.50000

AFIX 0

O3 4 0.259026 0.941521 0.403351 11.00000 0.04558 0.06638 =

0.05965 0.01401 0.00178 -0.00520

AFIX 147

H3 2 0.223899 0.873688 0.398937 11.00000 -1.50000

AFIX 0

N1 3 0.174047 0.640269 0.436854 11.00000 0.03982 0.06891 =

0.04527 -0.00297 0.01145 -0.00463

C1 1 -0.084457 0.315008 0.358959 11.00000 0.05266 0.08014 =

0.05906 -0.00184 0.00757 -0.01427

PART 1

AFIX 66

C2A 1 -0.015604 0.384091 0.376247 21.00000 0.04690 0.06474 =

0.04652 -0.00153 0.00729 -0.00396

C3A 1 0.029779 0.233770 0.425508 21.00000 0.04690 0.06474 =

0.04652 -0.00153 0.00729 -0.00396

AFIX 43

H3A 2 0.017819 0.079068 0.444734 21.00000 -1.20000

AFIX 65

C4A 1 0.093037 0.314671 0.446033 21.00000 0.04690 0.06474 =

0.04652 -0.00153 0.00729 -0.00396

AFIX 43

```
H4A   2    0.123401    0.214096    0.478992    21.00000   -1.20000
AFIX  65
C5A   1    0.110912    0.545892    0.417298    21.00000    0.04690    0.06474 =
         0.04652   -0.00153    0.00729   -0.00396
C6A   1    0.065530    0.696215    0.368037    21.00000    0.04690    0.06474 =
         0.04652   -0.00153    0.00729   -0.00396
AFIX  43
H6A   2    0.077490    0.850917    0.348811    21.00000   -1.20000
AFIX  65
C7A   1    0.002272    0.615317    0.347511    21.00000    0.04690    0.06474 =
         0.04652   -0.00153    0.00729   -0.00396
AFIX  43
H7A   2   -0.028092    0.715893    0.314552    21.00000   -1.20000
AFIX  66
PART 2
C2B   1   -0.017598    0.401650    0.382926   -21.00000    0.04690    0.06474 =
         0.04652   -0.00153    0.00729   -0.00396
C3B   1    0.022168    0.315738    0.452030   -21.00000    0.04690    0.06474 =
         0.04652   -0.00153    0.00729   -0.00396
AFIX  43
H3B   2    0.006099    0.208312    0.485645   -21.00000   -1.20000
AFIX  65
C4B   1    0.085952    0.390388    0.470891   -21.00000    0.04690    0.06474 =
         0.04652   -0.00153    0.00729   -0.00396
AFIX  43
```

```
H4B   2    0.112558    0.332907    0.517125   -21.00000   -1.20000
AFIX  65
C5B   1    0.109969    0.550949    0.420649   -21.00000    0.04690    0.06474 =
        0.04652   -0.00153    0.00729   -0.00396
C6B   1    0.070203    0.636862    0.351546   -21.00000    0.04690    0.06474 =
        0.04652   -0.00153    0.00729   -0.00396
AFIX  43
H6B   2    0.086272    0.744289    0.317931   -21.00000   -1.20000
AFIX  65
C7B   1    0.006420    0.562214    0.332683   -21.00000    0.04690    0.06474 =
        0.04652   -0.00153    0.00729   -0.00396
AFIX  43
H7B   2   -0.020186    0.619695    0.286449   -21.00000   -1.20000
AFIX   0
PART 0
C8    1    0.217390    0.540009    0.489857    11.00000    0.04530    0.06784 =
        0.05095    0.00403    0.01143   -0.00669
AFIX  43
H8    2    0.206455    0.408753    0.520575    11.00000   -1.20000
AFIX   0
C9    1    0.282409    0.623005    0.503633    11.00000    0.03666    0.06552 =
        0.04072   -0.00223    0.00909    0.00049
C10   1    0.301100    0.822179    0.458189    11.00000    0.04387    0.05138 =
        0.04099    0.00116    0.00278    0.00259
C11   1    0.365978    0.884838    0.471135    11.00000    0.03761    0.04873 =
```

0.04497 -0.00329 0.00716 -0.00164

C12 1 0.410388 0.767767 0.528551 11.00000 0.03741 0.05755 =

0.05240 -0.00183 0.00745 -0.00389

AFIX 43

H12 2 0.453215 0.814285 0.536611 11.00000 -1.20000

AFIX 0

C13 1 0.391454 0.579131 0.574863 11.00000 0.04476 0.05864 =

0.04620 0.00168 0.00248 0.00055

C14 1 0.328461 0.505518 0.560785 11.00000 0.05681 0.06829 =

0.03645 0.00665 0.01434 -0.00312

AFIX 43

H14 2 0.316472 0.373429 0.590405 11.00000 -1.20000

AFIX 0

MOLE 2

S1 5 0.255327 0.375973 0.202039 11.00000 0.06028 0.06068 =

0.05528 -0.00488 0.00787 0.01580

O4 4 0.213515 0.443582 0.123358 11.00000 0.06271 0.11691 =

0.04975 -0.00673 0.00487 0.03051

C15 1 0.208519 0.392843 0.272570 11.00000 0.07653 0.08846 =

0.05575 0.00320 0.01424 0.00106

AFIX 137

H15A 2 0.234683 0.357284 0.323515 11.00000 -1.50000

H15B 2 0.174689 0.268631 0.260502 11.00000 -1.50000

H15C 2 0.190561 0.561848 0.272309 11.00000 -1.50000

AFIX 0

C16 1 0.303203 0.647879 0.231446 11.00000 0.11954 0.09631 =

0.09254 0.01182 0.01277 -0.03983

AFIX 137

H16A 2 0.337971 0.649890 0.204937 11.00000 -1.50000

H16B 2 0.319851 0.641889 0.287121 11.00000 -1.50000

H16C 2 0.277934 0.800356 0.218342 11.00000 -1.50000

AFIX 0

HKLF 4 1 1 0 0 0 1 0 0 0 1

REM 4ABA35DIIODO in P2(1)/c ORANGE NEEDLE

REM wR2 = 0.1028, GooF = S = 1.008, Restrained GooF = 1.008 for all data

REM R1 = 0.0439 for 2516 Fo > 4sig(Fo) and 0.0865 for all 4128 data

REM 186 parameters refined using 0 restraints

END

WGHT 0.0334 0.5617

REM Instructions for potential hydrogen bonds

HTAB O2 S1_$1

HTAB O2 O4_$1

HTAB O3 N1

HTAB C6B O1_$2

HTAB C8 O4_$3

HTAB C12 I2_$4

HTAB C14 S1_$3

HTAB C15 O3_$5

HTAB C15 O1_$1

HTAB C15 O1_$2

REM Highest difference peak 0.868, deepest hole -0.754, 1-sigma level 0.105

Q1 1 0.4502 0.5351 0.6716 11.00000 0.05 0.87

Q2 1 0.4562 0.3355 0.6824 11.00000 0.05 0.73

Q3 1 0.4687 0.3340 0.6153 11.00000 0.05 0.68

Q4 1 0.3593 1.1709 0.3988 11.00000 0.05 0.65

Q5 1 0.4003 0.9926 0.3888 11.00000 0.05 0.62

Q6 1 0.4440 1.1881 0.4113 11.00000 0.05 0.61

Q7 1 0.4090 0.3643 0.6515 11.00000 0.05 0.59

Q8 1 0.4127 1.1438 0.4419 11.00000 0.05 0.50

Q9 1 -0.0182 0.5049 0.3711 11.00000 0.05 0.45

Q10 1 0.2445 0.6183 0.1957 11.00000 0.05 0.41

Q11 1 0.3359 0.1208 0.6326 11.00000 0.05 0.40

Q12 1 0.5435 0.4131 0.6509 11.00000 0.05 0.39

Q13 1 0.5075 0.3759 0.6691 11.00000 0.05 0.39

Q14 1 0.0458 0.6216 0.3386 11.00000 0.05 0.39

Q15 1 0.0011 0.3437 0.4091 11.00000 0.05 0.38

;
